# Evaluating the Performance and Implementation of the 2018 Classification of Periodontal Diseases: A Systematic Review and Survey

**DOI:** 10.1111/jcpe.14170

**Published:** 2025-05-07

**Authors:** Nicola X. West, Alexander Gormley, Alexander J. Pollard, Rossana Izzetti, Crystal Marruganti, Filippo Graziani

**Affiliations:** ^1^ Clinical Trials Unit, Periodontology Bristol Dental School, University of Bristol Bristol UK; ^2^ Università di Pisa Pisa Italy; ^3^ University College London London United Kingdom

**Keywords:** diagnosis, epidemiology, periodontal diseases, systematic review

## Abstract

**Aim:**

To evaluate the performance and implementation of the 2018 Classification of Periodontal Diseases for periodontitis through systematic review and survey methodology.

**Materials and Methods:**

A two‐part systematic review was conducted. Part 1 aimed, with descriptive statistics, to evaluate performance metrics of the 2018 Classification, including diagnostic accuracy, prognostic performance and examiner reliability. Part 2 used thematic analysis to elucidate facilitators and barriers to the implementation of the classification. A survey collected evidence of barriers, facilitators and local implementation.

**Results:**

Part 1 of this review included 14 individual studies of moderate risk of bias. Eight studies examined diagnostic accuracy, three studies examined prognostic efficacy and three studies examined inter‐/intra‐rater reliability. Part 2 included 33 individual studies with data considered at high risk of bias. The survey returned 1113 responses. The thematic analysis in Part 2 led to seven findings, five related to facilitators and two concerning barriers to the implementation. Survey results showed that 78% of respondents currently use the classification, but the most common concerns relate to its complexity.

**Conclusions:**

The 2018 Classification performs well as a classification in comparison with previous classifications. Specific identified barriers have potentially limited the comprehensive uptake of the classification.

## 1 Introduction

Periodontal diseases represent a range of chronic inflammatory conditions that affect the supporting structures of the teeth. Severe periodontitis is the sixth most prevalent disease globally, and estimates suggest it affects over 10% of the population worldwide (Kassebaum et al. 2014), and in more recent data, 13.1% (Nascimento et al. 2024). If not managed appropriately, this chronic non‐communicable disease leads to progressive loss of the periodontal supporting structures, with the clinical end point being tooth loss (Arias‐Bujanda et al. 2020; Chambrone et al. 2010). Periodontitis leads to adverse outcomes including discomfort or pain, nutrition, reduced social confidence, willingness to smile, well‐being, speech and overall quality of life (Garcia et al. 1998; Ide et al. 2016; Sanz, Marco Del Castillo, et al. 2020; Sharma et al. 2016; Soikkonen et al. 2000). Periodontitis is associated with multiple other chronic non‐communicable diseases, including but not limited to diabetes, chronic kidney disease, cardiovascular disease, dementia and, in some cases, premature mortality (Botelho et al. 2022). Periodontitis represents a significant public health concern, causing disability and being a source of social inequality. The financial cost of periodontitis has been estimated at $55 billion global productivity loss in 2015 (adjusted for purchasing power parity) (Righolt et al. 2018). Economist Impact (2021) showed that the economic benefit of increased prevention, diagnosis and management of periodontitis, led by patient home care, had a positive return on investment in six European countries investigated.

Providing a classification and diagnosis for periodontitis are the initial steps to successful treatment. Previous periodontal classification systems could be critiqued for overlapping disease categories, inappropriate emphasis on age of onset of disease and failure to properly recognise the rate of progression. Importantly, a case definition for periodontal health was provided for the first time in the new classification, with identification and recognition of oral health as a step change for periodontal care (Chapple et al. 2018). In addition, previous classifications did not recognise the stable periodontitis patient, an addition that constitutes further benefit for both clinicians and patients.

The new classification for the diagnosis of periodontal diseases and conditions was developed at the 2017 World Workshop on the Classification of Periodontal and Peri‐implant Diseases and Conditions and reflects the oral healthcare profession's improved understanding of periodontal diseases (Caton et al. 2018). The workshop was co‐sponsored by the European Federation of Periodontology (EFP) and the American Academy of Periodontology (AAP) and included global expert participants. There were four working groups focusing on different aspects of periodontal diseases: periodontal health and gingival diseases and conditions; periodontitis; periodontal manifestations of systemic diseases and acquired conditions; and peri‐implant diseases and conditions (Berglundh et al. 2018; Chapple et al. 2018; Jepsen et al. 2018; Papapanou et al. 2018).

The new classification aimed to create a unified approach to the classification of periodontal and peri‐implant diseases. Thus, it allows practitioners to classify and manage periodontal diseases consistently, understanding the nature of a patient's disease through diagnosis. Further emphasis was placed on the patient's rate of disease progression, rather than the age of onset, to focus spotlight on a patient's susceptibility to disease and consider an individual's combined risk‐factor profile. Conditions previously termed ‘chronic’ and ‘aggressive’ periodontitis were now simply recognised as periodontitis because of a lack of evidence to support separate disease aetiology or pathophysiology. The 2018 Classification defines health and captures and distinguishes the severity of periodontal tissue loss as well as susceptibility for periodontitis (Chapple et al. 2018; Lang and Bartold 2018).

For the new classification of periodontitis (Papapanou et al. 2018) to be successfully implemented, it is important that it is valid, reliable and reproducible and can be rapidly implemented in clinical practice. Over half a decade has passed since the 2018 Classification has been published, and this systematic review with narrative synthesis sets out to explore these factors.

The aim of this review is therefore

- to evaluate the performance and implementation of the classification of periodontal diseases according to the 2018 Classification of Periodontal Diseases with regard to only periodontitis (Papapanou et al. 2018).

The objectives are

- to evaluate the current (2018) classification for its accuracy, prognostic efficacy and reproducibility in classifying periodontal disease, and
- to identify the facilitators and barriers influencing the utility and implementation of the classification through systematic reviews and a cross‐sectional survey

## 2 Materials and Methods

This paper reports a mixed‐methodology study encompassing two interlinked systematic reviews and a cross‐sectional study survey. The systematic review protocol was devised and approved by the European Federation of Periodontology workshop committee in June 2024; the protocol was registered on the PROSPERO registry (CRD42024560707). The study is split into two parts to reflect the evaluation of the classification; each part will report a separate study methodology and results, and these will be discussed jointly.

### 2.1 Part 1: Performance of the Classification—Systematic Review

#### 2.1.1 Focused Question

How does the 2018 Classification of Periodontal and Peri‐Implant Diseases and Conditions perform as a classification metric compared with previous classifications?

#### 2.1.2 Search Strategy

A bespoke search strategy has been developed with specialist periodontists, general dentists, academic staff experienced in systematic review methodology and an information specialist. The strategy is provided in Appendix 1 and uses Medical Subject Headings (MeSH) and targeted keywords in conjunction with Boolean operators. Comprehensive searches were undertaken in OvidSP versions of Medline and EMBASE by A.G. The grey literature was searched, and citations of the 2018 Classification paper or those directly related to the 2017 World Workshop on the Classification of Periodontal Diseases and Conditions were hand‐searched (A.G., A.J.P., C.M. and R.I.). No limitations on language were placed on the search.

#### 2.1.3 Eligibility Criteria

Table 1 shows the eligibility criteria for Part 1 of this review.

**TABLE 1:** Part 1: Eligibility criteria.

	Inclusion criteria	Exclusion criteria
Participants	Specialist periodontists, general dentists, dental hygienists, dental therapists, dental care professionals, students of any aforementioned profession Human adult (18+) patients	Non‐dentally‐qualified health professional undertaking assessment
Index test	2018 Classification (including local implementations of the classification, which explicitly stated the 2017 World Workshop classification as the source)	Previous classifications of periodontitis
Condition	Periodontitis as defined by the European Federation of Periodontology (Papapanou et al. 2018)	Periodontal health Gingivitis Peri‐implant disease Non‐plaque‐related periodontal disease
Type of studies	Diagnostic test accuracy study Longitudinal study Cross‐sectional study	Pre‐clinical, animal or laboratory studies
Outcomes	Performance measure of the 2018 Classification including ○ Inter‐rater outcomes (agreement and kappa scores) ○ Diagnostic accuracy (sensitivity and specificity values) ○ Predictive ability (any prognostic study where the 2018 Classification or its implementation has been explicitly used as the source and the outcome of tooth loss is assessed).	Descriptive outcomes Qualitative outcomes Outcomes related to inter‐rater assessments, diagnostic accuracy or predictive ability, but do not specifically report the values as stated in the inclusion criteria.

#### 2.1.4 Data Management

Citations were collated in EndNote X9 following the searches. De‐duplication was done using automatic software functions. De‐duplicated articles were uploaded to the Rayyan systematic review web application (https://www.rayyan.ai/); a secondary de‐duplication exercise was undertaken, followed by initial abstract screening. Two blinded reviewers (A.G. and R.I.) independently screened the abstracts against the pre‐defined eligibility criteria. Conflicts were resolved with consensus discussions among all authors. Included abstracts then taken to the next stage of full‐text screening. This blinded process was again completed independently, and any conflicts were discussed for consensus. Full‐text records that, following screening, were deemed ineligible for inclusion are listed in Appendix 3, along with the reason for ineligibility.

#### 2.1.5 Data Extraction and Synthesis

A data extraction form was developed, initially piloted for three studies, by A.G. and R.I., which, following consensus approval, was then used for each included study with double‐blinded extraction. The data extraction form is available in Appendix 5.

The initial stage involved a descriptive analysis of the included studies to determine the types of classification evaluation studies conducted, the availability of data and the characteristics of the studies. At this stage, an assessment was made regarding the inclusion of a meta‐analysis. Considerable heterogeneity was evident in the study designs, populations, study characteristics and populations investigated in the included studies, so a consensus decision was made to conduct a narrative synthesis. This proceeded through the use of the developed evidence tables presented in this review. The synthesis was guided by the overall domain of investigation in each study; the domains identified included diagnostic accuracy (studies that directly evaluated the 2018 Classification against any other described classification through diagnostic accuracy assessments reporting sensitivity and specificity values), prognostic efficacy (studies that compared the 2018 Classification and any other defined classification in a cohort study to assess its performance in the prediction of tooth loss related to periodontal disease) and examiner reliability (studies that assessed the reliability of examiners in classifying cases using the 2018 Classification). The data extraction table is presented in Appendix 5.

#### 2.1.6 Quality Appraisal

Validated quality appraisal tools were used to appraise each included study blindly and with double assessment by A.G. and R.I. Studies reporting diagnostic accuracy were assessed using the QUADAS‐2 (Quality Assessment of Diagnostic Accuracy Studies) tool, prognostic models with the PROBAST (Prediction model study Risk Of Bias Assessment Tool) and examiner reliability with the QAREL (Quality Appraisal of Reliability Studies) tool.

### 2.2 Part 2: Implementation of the Classification—Systematic Review

#### 2.2.1 Focused Question

What are the barriers and facilitators to the implementation of the 2018 Classification of Periodontal and Peri‐Implant Diseases and Conditions, specifically in relation to the classification of plaque‐induced periodontal diseases?

#### 2.2.2 Search Strategy

This is described in Section 2.1.2 and available in Appendix 2. No limitations on language were placed on the search.

#### 2.2.3 Eligibility Criteria

Table 2 shows the eligibility criteria for Part 2 of this review.

**TABLE 2:** Part 2: Eligibility criteria.

	Inclusion criteria	Exclusion criteria
Population	Dental health professionals (including students) or patients Epidemiologists or research scientists	Non‐dentally‐qualified health professionals
Phenomena of interest	2018 Classification (including local implementations of the classification, where it explicitly stated the 2017 World Workshop classification has been the source).	Previous classifications of periodontitis
Context	Clinical care Oral epidemiological studies	
Type of studies	Any study design or written guidance issued by EFP member societies	Pre‐clinical, animal or laboratory studies

The synthesis will include any published article in a peer‐reviewed journal that reports barriers and/or facilitators to implementing the 2018 Classification in any section of the report. It may formally explore the topic as a primary or secondary objective, or the authors may make comments in any section of the paper related to perceived barriers or facilitators.

#### 2.2.4 Data Management

This is as described in Section 2.1.4, but this part was undertaken by A.J.P. and C.M.

#### 2.2.5 Data Extraction and Synthesis

Appendix 4 lists studies excluded from the review at the full‐text stage, along with the reasons for exclusion. Data were included from any part of a paper if it related to the new classification; this included quotes, themes, subthemes, interpretations made by study authors in relation to study data (qualitative or quantitative) or anything else that the reviewers felt relevant. The data extraction form is available in Appendix 6, which was piloted and refined prior to data collection. Data extraction was conducted by two reviewers to ensure nothing relevant was omitted.

A narrative synthesis was performed where identified influencing factors were grouped according to facilitator or barrier status. Other themes were considered if they arose once the reviewers were familiar with the dataset. Initial scoping searches showed that the data were likely to be limited and descriptive as opposed to highly theorised or conceptual. This proved to be true, so a thematic approach was considered appropriate for the analysis and synthesis of evidence. A reflexive thematic analysis (TA) approach was used aiming to produce a robust analysis (Braun and Clarke 2006). Two reviewers began by coding in parallel, subsequently meeting to discuss the codes to derive a consensus. This process continued until all relevant data were coded, and if there was doubt about the relevance of the data despite discussion, they were re‐coded. Themes were developed along with relevant findings.

#### 2.2.6 Quality Appraisal

Usually a ‘Risk to rigour’ assessment would be performed for a qualitative evidence synthesis to determine confidence of the methodology used in the included studies. However, there were no qualitative studies that met the review criteria, and many different types of articles were considered in order to gather opinions on the new classification. The authors have attempted to take a systematic approach to selecting the papers and analysing the data to reduce the likelihood of their own biases influencing the results. However, it should be considered that all data in this part of the review are simply the opinion of the included article's author/s and should be considered to be at high risk of bias. Nonetheless, examining the perception of the 2018 Classification since its introduction is necessary given that it is a live classification system, to be updated as new evidence emerges.

#### 2.2.7 Review Author Reflexivity

In line with quality standards, the reviewers sustained reflexivity throughout all stages of this review. Consideration was given as to how the authors' experiences and beliefs could influence the choices made regarding the review methods and interpretation of the data. The review authors work primarily in secondary care and academic settings related to periodontics, which may have influenced their interpretation of the data. However, this clinical experience may also have given a greater ability to understand the themes that developed. The authors continually reflected on potential strain between the perspectives of clinicians, academics and commissioners of healthcare.

### 2.3 Survey

A survey was developed to capture evidence of local implementation of the classification. The survey was distributed through the EFP MailChimp database, which includes details of the majority of the EFP National Society members, anyone who has registered for the EFP Newsletter and anyone who has registered for an EFP event. The survey is presented in Appendix 14 of Supporting Information S4.

#### 2.3.1 Eligibility Criteria

Any dental professional (including students) from any country with membership to a periodontal society affiliated with the EFP (the respondent is not required to be a member of their respective society) was deemed eligible.

#### 2.3.2 Survey Validation

A validation exercise was undertaken with a pilot of the survey distributed to a variety of stakeholders including a periodontist, a general dentist, a dental care professional and a student. Written feedback was sought following the completion of the pilot in order to refine and adapt the survey and its presentation where required. Specific queries included checking for common errors such as double‐barrelled, confusing or leading questions. An EFP steering committee subsequently reviewed and approved the final version.

#### 2.3.3 Survey Distribution

Responses were collated anonymously through Microsoft Forms by email (MailChimp) using the EFP central communications system and through the EFP Newsletter; reminder emails were sent to encourage a strong response rate. The survey was open between 28 June and 19 July 2024. Real‐time analysis of demographic profiles of responses was undertaken and monitored so that targeted reminders could be sent to groups or countries where responses were limited. The EFP social media pages were also used to share the survey link.

#### 2.3.4 Analysis of Survey Results

All responses to the survey were anonymous with some basic demographic data collected for context. The results were manually ‘cleaned’ to remove any spurious or accidental responses, as well as duplications. Any responses that appeared unusual were reviewed by a second author and, if it was felt that a response could be fictional or details were too similar to another entry, indicating possible duplication, it was deleted. Descriptive statistics regarding survey completion and demography of participant are provided. It was intended that a thematic analysis would be undertaken for open‐ended questions, but the responses tended to be so brief that a decision was made to simply describe overarching themes within the data. The ‘analysis’ was performed by two reviewers with feedback meetings with a senior member of the team. The response rate was calculated, with consideration given to how this affects the validity of results.

## 3 Results

### 3.1 Part 1: Performance of the Classification—Systematic Review

#### 3.1.1 Study Selection

**FIGURE 1:**
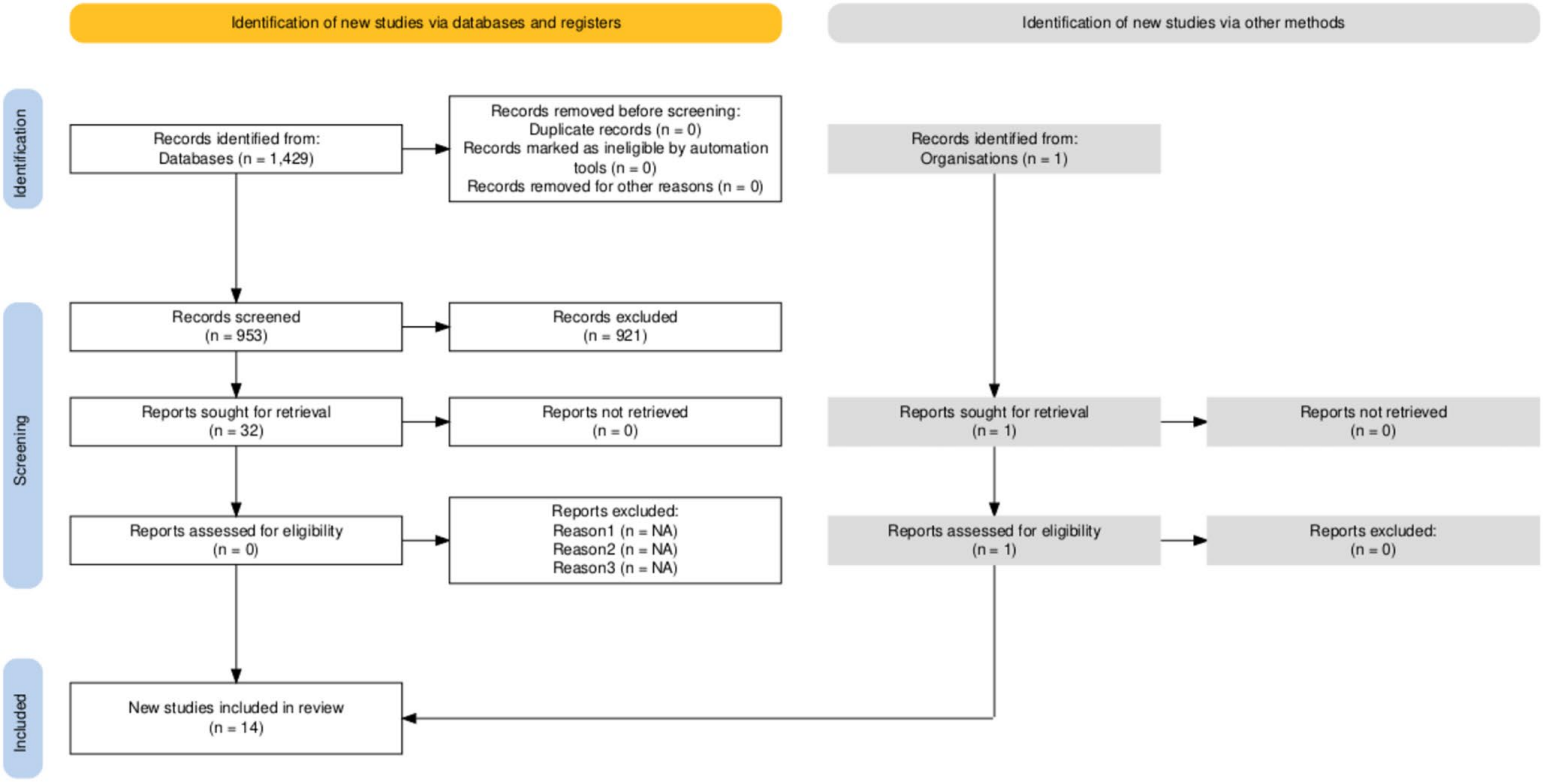
PRISMA diagram, as generated by Haddaway et al. (2022).

The searches were undertaken on 23 June 2024. A total of 1429 citations were obtained; following de‐duplication, 953 abstracts were screened and 32 full texts were retrieved for screening. An additional record was obtained from hand‐searching. The review contained 14 studies from 14 publications that fulfilled the eligibility criteria (see Figure 1). The majority of the studies (*n* = 8) assessed an element of diagnostic accuracy, assessing the 2018 Classification against a reference standard in a number of defined sub‐population groups. Prognostic efficacy was evaluated in three studies with the outcome of periodontal‐related tooth loss under investigation, and variations of examiner reliability (inter and intra) assessments were performed in three studies.

#### 3.1.2 Study Characteristics

The main characteristics from each included study are presented in Table 3. As part of the data synthesis, the studies were grouped on the domain of classification evaluation that was undertaken; the groupings of diagnostic accuracy, prognostic efficacy and examiner reliability were selected based on the characteristics of the included studies, and the narrative synthesis proceeded between these groups.

**TABLE 3:** Summary characteristics of included studies.

Authors	Year of publication	Number of participants	Study design	Statistical analysis used
Abrahamian et al.	2022	7 + 174	Cross‐sectional	Kappa agreement analysis
Botelho et al.	2020	6940	Cross‐sectional	Diagnostic test accuracy
Brito et al.	2021	243	Cross‐sectional	Diagnostic test accuracy
Costea et al.	2022	141	Cross‐sectional	Diagnostic test accuracy
Dukka et al.	2022	270	Retrospective cohort	Multivariate regression modelling
El Sayed et al.	2022	79	Retrospective cohort	Receiver operating characteristic (ROC) curve analysis
Li et al.	2022	204	Cross‐sectional	Diagnostic test accuracy
Machado et al.	2020	456	Cross‐sectional	Diagnostic test accuracy
Marini et al.	2020	30	Cross‐sectional	Kappa agreement analysis
Morales et al.	2022	1456	Cross‐sectional	Diagnostic test accuracy
Ortigara et al.	2020	588	Cross‐sectional	Diagnostic test accuracy
Ravidà et al.	2021	103	Cross‐sectional	Kappa agreement analysis
Saleh et al.	2021	167	Retrospective cohort	Multivariate Cox regression frailty analysis
Shi et al.	2023	435	Cross‐sectional	Diagnostic test accuracy

#### 3.1.3 Diagnostic Accuracy

The accuracy of the classification as a diagnostic tool was assessed in eight of the included studies. Reporting at the level of stage, grade and extent was poor, with the majority of studies reporting using the presence of periodontitis or the composite periodontal diagnosis as reported in Appendix 7 of Supporting Information S1. Three studies (Brito et al. 2022; Morales et al. 2022; Ortigara et al. 2021) assessed the diagnostic performance of the 2018 Classification against the reference standard of the 2012 AAP/CDC classification; the sensitivity of the classification was regarded as high, although its specificity reported was low. The accuracy of the classification was examined (Costea et al. 2022; Li et al. 2022) in a subset population of ischaemic stroke patients and pregnant women, respectively. Both the aforementioned studies refer to the highly sensitivity of the 2018 Classification in detecting even mild periodontal disease. Partial diagnostic screening indices were assessed by two studies (Shi et al. 2024; Botelho et al. 2020). Both evaluated Ramfjord teeth, while Botelho et al. (2020) also examined a number of other partial diagnostic protocols against the 2018 Classification and in comparison with the 2012 CDC/AAP Classification. Both studies found that the 2018 Classification outperformed previous classifications with respect to the diagnosis and staging of periodontal diseases, which the respective authors believe strengthens the potential of these screening protocols especially in the epidemiological context. The assessment of radiography‐based periodontal bone level as a screening tool for periodontitis using panoramic radiography was examined by Machado et al. (2020), who compared the method with a clinical periodontal examination aligned to both the 2018 and 2012 Classifications; the result showed a higher performance when used with the 2018 Classification.

#### 3.1.4 Prognostic Efficacy

The prognostic capability of the classification with regard to tooth loss was assessed in El Sayed et al. (2022), Dukka et al. (2022) and Saleh et al. (2022) in the review. A summary table is provided in Appendix 8 of Supporting Information S1. All studies used retrospective cohort study designs; the El Sayed et al. (2022) paper used logistic regression modelling including the 2018 or the 1999 Classification as the independent influencing variable. The subsequent AUC curve was calculated for each classification. No significant difference could be detected between the classifications for the prediction of tooth loss. Dukka et al. (2022) compared the British Society of Periodontology and Implant Dentistry UK implementation (BSP‐I) of the classification and the original 2018 Classification. The outcome of tooth loss due to periodontal‐related causes was assessed with the prognostic performance measured by the Harrell‐C index in comparing the model's prognostic stratification performance. For the BSP‐I, all variables (stage, grade and extent) correlated significantly with tooth loss due to periodontitis in both univariate and multivariate analyses, and in the 2018 International Classification, stages III and IV periodontitis correlated significantly with a worse prognosis. Marginal difference was recorded between the models, with both the 2018 Classification and the BSP‐I of the new classification reporting excellent predictive ability. Saleh et al. (2022) reported a prognostic study evaluating the long‐term performance of four Periodontal Risk Assessment Tools (PRATs), namely PerioRisk, periodontal risk assessment, periodontal risk calculator and staging and grading systems. In this study, the staging and grading systems correspond to the updated 2018 Classification. The study validated a comparison between periodontal and non‐periodontal related tooth loss in a single centre, within each risk class, and using Cox proportional hazard regression modelling. All PRATs performed well, and figures for Harrell's C index ranged from 0.62 to 0.67, with PerioRisk performing the best.

#### 3.1.5 Intra‐/Inter‐Examiner Reliability

Three studies assessed elements of inter‐ and intra‐rater reliability related to the utilisation of the classification (Appendix 9 of Supporting Information S1). All three studies employed a cross‐sectional examiner reliability type methodology. The geographical spread of studies encompassed Europe and the United States. The studies were published between 2020 and 2022. Two studies (Marini et al. 2021; Ravida et al. 2021) used a reference standard of a classification author(s) involved in the 2017 workshop, while Abrahamian et al. (2022) conducted an intra‐rater reliability assessment between seven ‘classification experts’ and a consensus setting exercise. Studies reported a variety of concordances/agreements and calculated kappa values for individual diagnostic elements (e.g., stage, grade and extent) and for a composite diagnosis.

For the purpose of this review, the kappa values have been interpreted using the method of Altman and Altman (1999), although varying interpretations of these values are possible. Ravida et al. (2021) reported overall moderate agreement (0.48–0.51) between their rater panel and the reference standard, albeit with wide confidence intervals. Agreement between the ‘classification experts’ in Abrahamian et al. (2022) was moderate to very good (0.52–0.85), although concordance between the individual raters and reference panel was weaker, while Marini et al. (2021) reported fair to moderate agreement (0.37–0.48) across individual diagnostic components. Authors of all studies noted that moderate concordance in agreement was possible within the remit of this classification; further refinements would enhance consistency and accuracy, especially in borderline cases.

No paper included in this review reported on the role of patient and/or public involvement in the design, conduct or dissemination of the study.

#### 3.1.6 Quality Assessment

Diagnostic accuracy studies were assessed with the QUADAS‐2 tool. The full results are available in Appendix 10 of Supporting Information S2. The majority of the studies were deemed to have a low risk of bias; however, owing to the populations included, their overall applicability to the review question was not always clear.

Studies reporting prognostic efficacy were assessed using PROBAST. The three included studies were all evaluated with a low reported risk of bias (Appendix 11 in Supporting Information S2), and both concluded high applicability to the populations under investigation.

In the three included examiner reliability assessments, the QAREL tool was used for quality assessment. All three studies performed well (Appendix 12 in Supporting Information S2), although the ordering of the cases provided for assessment was not clear or not varied in their presentation to the examiners.

### 3.2 Part 2: Implementation of the Classification—Systematic Review

#### 3.2.1 Study Selection

**FIGURE 2:**
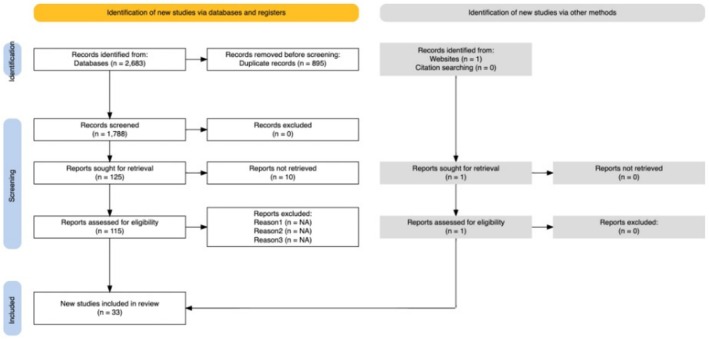
PRISMA diagram, as generated by Haddaway et al. (2022).

The searches took place on 23 June 2024, yielding 2683 records, which led to 1788 abstracts screened after de‐duplication. From these, 125 progressed to full text review. Thirty‐two of these studies were included in this review, as was one further study, which had been retrieved and excluded as a conference abstract but was later published in full text and a decision was made to include the commentary article (Raittio et al. 2025). Appendix 16 of Supporting Information S6 provides details of the full‐text shortlisting decisions. As a result, 33 studies were included in this review (Aboalsaud et al. 2023; Alawaji et al. 2022; Babay et al. 2019; Botelho et al. 2020; Claydon et al. 2022; Costea et al. 2022; Du et al. 2023; Dukka et al. 2022; El Sayed et al. 2022; Ertas et al. 2023; Fidyawati et al. 2024; Gandhi et al. 2022; Germen et al. 2021; Graham and Turner 2020; Guler Ayyildiz et al. 2024; Holtfreter et al. 2024; Jayawardena et al. 2021; Karaaslan et al. 2021; Ke et al. 2023; Lee et al. 2022; Malmqvist et al. 2024; Marini et al. 2023; Meir et al. 2023; Miyamoto et al. 2019; Parsegian et al. 2021; Patel et al. 2022; Raittio et al. 2025; Sanchez‐Otalvaro et al. 2022; Schwendicke et al. 2020; Steigmann et al. 2021; Sutthiboonyapan et al. 2020; Tokede et al. 2024; Winkler et al. 2022). The reasons for exclusion of studies at the abstract level are detailed in the PRISMA diagram (see Figure 2). The reasons for exclusion of studies based on full‐text review are outlined in Appendix 4.

#### 3.2.2 Thematic Analysis

Two themes developed with a total of seven findings, which are outlined below with representative quotes. Appendix 13 of Supporting Information S3 reports the full codebook generated for this review, and Appendix 15 of Supporting Information S5 provides the raw data extraction and primary codes.

##### 3.2.2.1 Theme 1: Factors That Facilitate the Implementation of the 2018 Classification

Finding 1. *A number of clinical advantages were noted in relation to the new classification, particularly in relation to the personalised nature of staging and grading with incorporation of risk factors, the emphasis on early detection of periodontitis, the definition of health and stability and the prognostic value of the classification system*.

Multiple sources felt that the new classification promoted personalised diagnosis and treatment planning (Aboalsaud et al. 2023; Babay et al. 2019; Claydon et al. 2022; Karaaslan et al. 2021; Miyamoto et al. 2019; Schwendicke et al. 2020). Aboalsaud et al. (2023) recognised the importance of incorporating risk factors into the new system and commented that this helps to ‘… estimate the aggressiveness of the disease and takes into account individual factors that will likely impact the response to treatment and its desired outcomes’. Several sources commented that the incorporation of risk factors and alignment with the medical model facilitated inter‐professional collaboration when managing periodontitis patients (Aboalsaud et al. 2023; Claydon et al. 2022; Holtfreter et al. 2024). The logical next step using the new classification, according to Babay et al. (2019), is to provide ‘… tailor‐made treatment plans for every patient’.

Another perceived clinical advantage was that the change in case definition allows earlier detection of periodontitis, facilitating early referral and active preventative strategies (Costea et al. 2022; Germen et al. 2021; Karaaslan et al. 2021; Miyamoto et al. 2019). For example, Costea et al. (2022) felt that ‘The high sensitivity value underlines the capacity of the new case definition system to detect periodontitis, including mild periodontal destructions, which greatly favours early treatment’. In addition, the recognition of health for the first time, along with clearly determined clinical end points, was seen as an advantage for clinicians when planning treatment and recall intervals. According to Claydon et al. (2022), this ‘… acts as an objective target for clinicians, which empowers patients to contribute to treatment planning and recall interval’. On the subject of recall, Miyamoto et al. (2019) reinforced the importance of grading, describing it as ‘critical for planning prevention of periodontal disease and indicates the need for meticulous periodontal maintenance’.

Several sources felt that the new classification was useful for prognosticating treatment outcomes and tooth loss (Aboalsaud et al. 2023; El Sayed et al. 2022; Miyamoto et al. 2019; Schwendicke et al. 2020). For instance, tooth loss was found to be ‘… associated with stage and grade (more teeth were lost in stage IV than III and grade C than B) …’ (Schwendicke et al. 2020).

Finding 2: *Several advantages were noted for use of the new classification in epidemiological studies, particularly in relation to the correlation with tooth loss and cost effectiveness, inclusion of mid‐buccal and lingual sites in partial‐mouth protocols and sensitivity for detecting disease even with partial diagnostic protocols*.

Schwendicke et al. (2020) mention the correlation between tooth loss and treatment costs as being an advantage of the new classification for epidemiological use. They state: ‘we found tooth loss to be associated with stage and grade (more teeth were lost in stage IV than III and grade C than B), while costs were rather driven by the grade (higher costs in grade C than B)’ (Schwendicke et al. 2020). Holtfreter et al. (2024) agreed that the new classification can reliably predict tooth loss and reported that it had ‘… lower susceptibility to bias as compared to the Centers for Disease Control and Prevention/American Academy of Periodontology (CDC/AAP) classification when partial‐mouth recording protocols are used’.

When considering the use of the new classification with partial‐mouth protocols, Botelho et al. (2020) felt that protocols that include buccal surfaces increased ‘… likelihood of correctly diagnosing periodontitis’ as well as having an ‘… improved ability to transmit the entire periodontal condition’.

Finding 3: *Implementation strategies such as the use of flowcharts were felt to be facilitators to the use of the new classification*.

Several sources felt that the use of flowcharts was successful in aiding correct staging and grading in cases of periodontitis (Ertas et al. 2023; Parsegian et al. 2021; Sutthiboonyapan et al. 2020). Ertas et al. (2023) used simple decision flowcharts to ‘… facilitate the performance of fast and accurate periodontitis staging and grading, but also to minimize confusion and inconsistent diagnoses’. Some reduced the steps necessary, commenting that ‘In this flowchart, clinical AL measurement may be skipped in some cases or it can be done only when necessary’ (Sutthiboonyapan et al. 2020).

Finding 4: *A number of local implementation strategies were discussed in the literature. These acted as facilitators for the implementation of the new classification. Some of the local implementation strategies had slight variations in their interpretation of the new classification*.

Sources noted that local implementation strategies with slight variations in their interpretation of the new classification had been adopted in the United Kingdom, United States, The Netherlands and Ireland (Jayawardena et al. 2021). The only strategy that was described in detail within the included papers was that of BSP‐I (Claydon et al. 2022; Dukka et al. 2022; Gandhi et al. 2022; Graham and Turner 2020; Jayawardena et al. 2021). In their discussion, Claydon et al. (2022) determined that the results of their study ‘indicate that application of the BSP implementation of the [2018] classification is attainable in the general dental practice operational framework, within a 1‐year timeframe’. The authors attributed this partly to the BSP, including ‘the great efforts made by the BSP to widely distribute educational material regarding the classification, aided by the uptake of social media in the dental profession’ (Claydon et al. 2022). They commented that ‘Such an implementation was arguably impossible at the time the 1999 Classification was released’ (Claydon et al. 2022).

As part of their dissemination campaign for the BSP‐I, the BSP produced flowcharts, webinars and multiple published case studies (Claydon et al. 2022; Gandhi et al. 2022; Graham and Turner 2020; Jayawardena et al. 2021). It was noted that the BSP‐I was a reductionist model of the original 2018 Classification but that this ‘neither affected the class allocation nor the prognostic performance of the system’ (Dukka et al. 2022).

Finding 5: *Several advantages were noted for the use of technological implementation strategies to facilitate the use of the new classification*.

Several sources felt that technological strategies could help automate/semi‐automate the classification/diagnosis process (Ertas et al. 2023; Fidyawati et al. 2024; Guler Ayyildiz et al. 2024; Lee et al. 2022; Malmqvist et al. 2024; Meir et al. 2023; Patel et al. 2022; Sanchez‐Otalvaro et al. 2022). Fidyawati et al. (2024) describe that ‘Artificial intelligence intends to reproduce the cognitive processes of the human being and obtain the same result, in this case, the determination of the diagnosis of a disease with that produced by the clinician, with more accuracy and shorter time’. Other authors suggest that staging is a challenging process because it contains ‘… many parameters’ and computer‐aided diagnostics can be used to provide a ‘second opinion’ when classifying diseases (Guler Ayyildiz et al. 2024). In fact, Guler Ayyildiz et al. (2024) report that ‘… deep learning (DL) shows high accuracy and perfect reliability in diagnosing periodontal bone loss and in staging periodontitis’. Surveys have been undertaken to assess how digital tools are perceived by target end users. One survey found that 80% of dental hygienists were positive about a digital tool to ‘facilitate and improve decision making’ (Malmqvist et al. 2024). Users of an app‐based classifier found that the questions within the app ‘… were very specific about periodontal parameters that define the periodontal condition and therefore improved their understanding of the new classification system’ (Sanchez‐Otalvaro et al. 2022).

##### 3.2.2.2 Theme 2: Factors Acting as Barriers to the Implementation of the 2018 Classification

Finding 6: *Several practical barriers to the implementation of the new classification were noted, such as the complexity of the classification, perceived subjectivity, especially with classifying borderline cases, and concerns about inter‐rater reliability*.

Within a university setting, some concerns were noted about ‘… lack of faculty buy‐in, lack of information and CE courses covering the new staging and grading system and no time in the curriculum for implementation’ (Aboalsaud et al. 2023). In addition, during the initial phase in of the classification, there was the ‘… challenge of introducing the new system while also still teaching the 1999 classification system because the [examining board] will not be testing on the new staging and grading system until the fall of 2021’ (Aboalsaud et al. 2023). Gandhi et al. (2022) felt that it was ‘… challenging for dental students to consider all the factors involved in an accurate periodontal diagnosis’.

In the practice setting, Ertas et al. (2023) described that the complexity of the new classification could lead to difficulties applying it to daily practice. For instance, they report that ‘… many clinicians have complained about difficulties in determining the stage and grade of periodontitis because of the presence of many clinical and radiographic factors…’ (Ertas et al. 2023). Guler Ayyildiz et al. (2024) agreed that ‘The new periodontal disease classification is a complex process…’. There were some concerns about there being ‘… several subjective factors that go into formulating a periodontal diagnosis…’ (Gandhi et al. 2022). Steigmann et al. (2021) agreed, stating that ‘Borderline cases require clinicians to additionally rely on their clinical judgement to overcome strict algorithmic assessments outside of the parameters of the general guidelines in order to arrive at an appropriate diagnosis’.

Finding 7: *Several barriers were noted in relation to the implementation of the new classification for the purpose of epidemiological studies and public health strategy. Concerns focused on the need for full‐mouth clinical and radiographic examination, the need to undertake a thorough clinical and medical history and the changing case definition leading to a potential overestimation of disease prevalence and subsequent risks this creates*.

A number of sources described barriers to the implementation of the classification in epidemiological studies (Alawaji et al. 2022; Botelho et al. 2020; Costea et al. 2022; Du et al. 2023; Germen et al. 2021; Graham and Turner 2020; Holtfreter et al. 2024; Karaaslan et al. 2021; Ke et al. 2023; Malmqvist et al. 2024; Raittio et al. 2025; Schwendicke et al. 2020). Alawaji et al. (2022) stated that ‘grading is less reliable without a full‐mouth radiographic assessment, which is not routinely conducted or recommended for epidemiological studies’ and that previous studies either ‘… adopted staging only for population‐based data…’ or acknowledged ‘… the challenges of using the full matrix of staging, grading, and descriptors of distribution’. Du et al. (2023) acknowledged that the new classification ‘… allows comprehensive assessment’ but note that it ‘… requires full‐mouth inspection, which can be time‐ and labour‐intensive in population‐based surveys and epidemiological investigations’. A further barrier is described by Holtfreter et al. (2024) who suggest that determining the reason for tooth loss can be challenging and the ‘… number of missing teeth due to periodontitis cannot be reliably determined’. They noted that ‘the few epidemiological studies applying the 2018 system so far have largely excluded tooth loss data from the assessment of stage’ and those that included tooth loss ‘… predominantly used assumptions rather than primary data’ (Holtfreter et al. 2024). Certain other complexity factors used in staging are not routinely collected, meaning that there is ‘… potential underestimation of the true prevalence of stage III and IV periodontitis’ (Holtfreter et al. 2024).

It was noted that differing case definitions between previous classifications and the new 2018 Classification meant that there may be differences in ‘… periodontitis identification and prevalence values’ which may limit ‘… direct comparison between studies’ (Costea et al. 2022). Costea et al. (2022) discuss that the new classification ‘… lowers the clinical attachment level (CAL) diagnostic threshold… which may increase the prevalence of periodontitis’. An increased prevalence of periodontitis was also documented by Germen et al. (2021), who felt that it was difficult to distinguish stage I periodontitis from gingivitis. One source found that ‘previously published epidemiological surveys… encountered additional difficulty in the distinction between gingivitis and periodontitis, as none of them reported the presence of gingivitis cases despite the inclusion of young individuals’ (Holtfreter et al. 2024). Raittio et al. (2025) expressed strong concerns about the new classification, remarking that ‘its development was not based on a balanced assessment of the potential benefits and harms associated with its implementation’. The authors were concerned that use of the new classification for epidemiological purposes leads to ‘… exorbitantly large prevalence estimates … that do not align well with the public health importance of periodontitis, and overdiagnosis is likely to be a considerable problem leading to an unfavourable benefits‐to‐harms ratio for major sections of any given population’ (Raittio et al. 2025). The authors suggest that the increased sensitivity of the classification may partly explain the increase in public spending in Norway ‘… for the cause‐related treatment of periodontitis from 21.1 million Euros in 2013 (before the introduction of the new classification system) to 53.6 million Euros in 2022 (after the introduction), representing an increase of 83%. These figures are deflated by the consumer price index for dental services. The increase without adjusting for inflation is 154%’ (Raittio et al. 2025). Raittio et al. (2025) raised concerns about the new classification as ‘… shift the benefits‐to‐harms ratio for periodontitis treatment in a negative direction for both patients and for society at large’, calling for a ‘… re‐evaluation of disease definitions… and treatment thresholds… aiming to attain levels that are both practically and financially feasible and relevant for patient‐important outcomes’.

Du et al. (2023) expressed the opinion that the new classification was better at distinguishing periodontitis cases, but that the 2012 CDC/AAP definition was more effective at identifying the population free from disease. They went on to suggest that this meant the new classification may be better for clinical use, whereas the CDC/AAP 2012 definition may be better used for surveillance studies in the future (Du et al. 2023).

The studies included in this review used differing interpretations of the new classification, including attempting to use it with full‐mouth partial diagnostic protocols (Botelho et al. 2020). Holtfreter et al. (2024) stated that ‘A concrete framework on how to apply the 2018 scheme to epidemiological data has not been provided in the original publication or consensus reports, allowing discrepancies due to different interpretations’.

### 3.3 Survey

#### 3.3.1 Distribution

The survey was sent to 17,511 individuals on the 28 June 2024, followed by a reminder (sent on 5 July) to 17,383 who had not clicked the link in the initial email. A second reminder email was sent on 16 July using analytics to target recipients who had not opened the first reminder; this went to 7155 recipients. The survey link was also shared on the EFP Instagram account. On 1 July, it had received 816 views (58 clicks), and on 8 July it had 2859 views (84 clicks).

**FIGURE 3:**
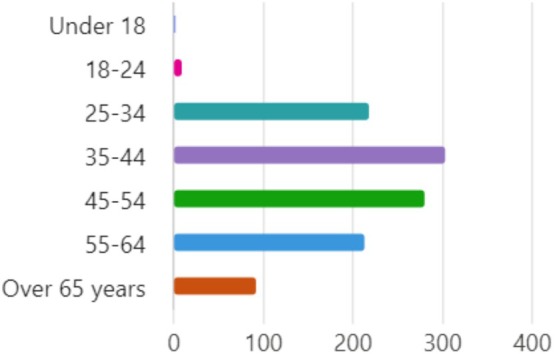
Age of respondents.

One‐thousand one‐hundred and thirty individual responses were received, giving a response rate of 6.4%. Figure 3 shows the age of the respondents (*n* = 1110 responses), showing a positively skewed distribution as expected.

**FIGURE 4:**
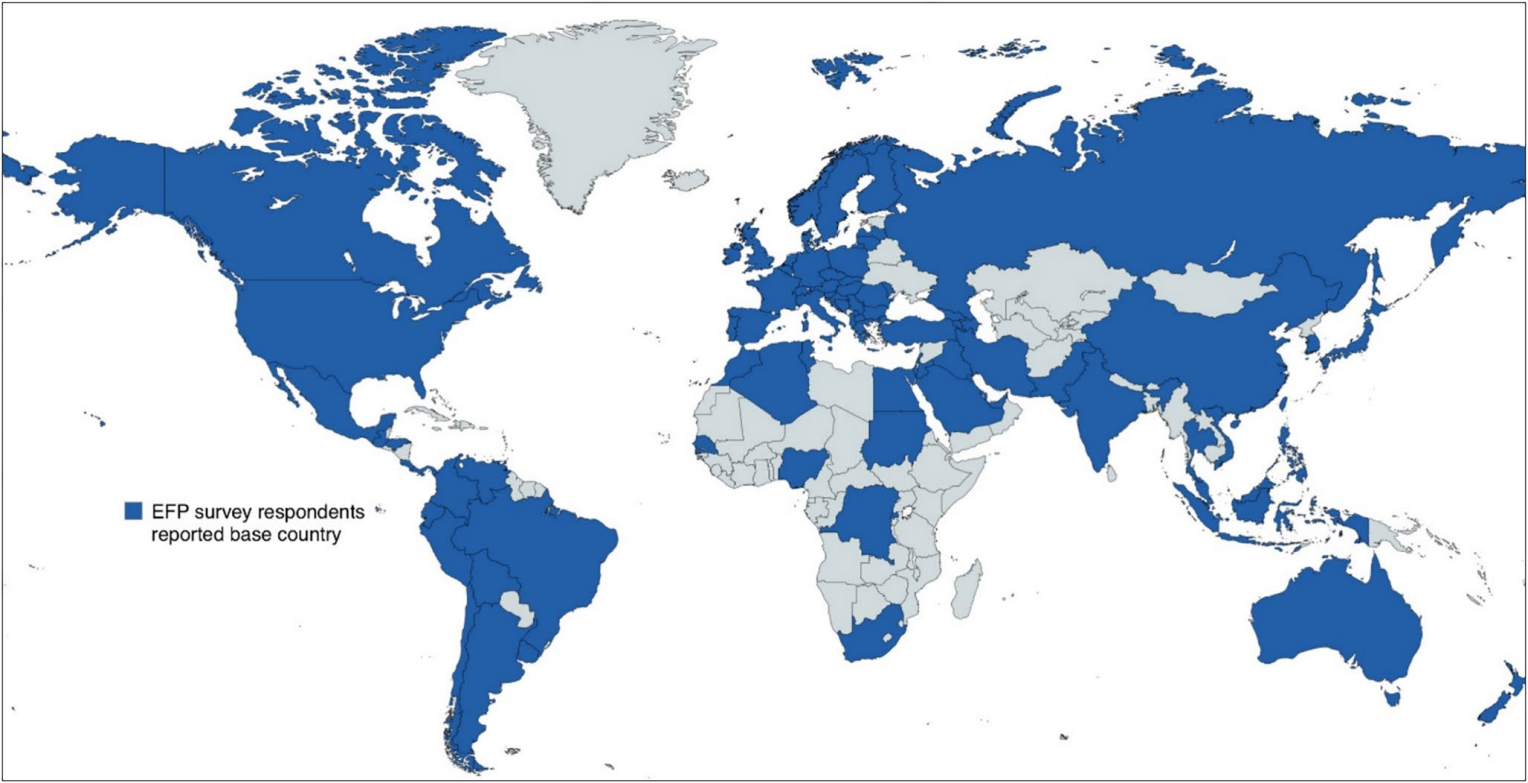
Respondent's reported base country.

Response came from 95 countries (Figure 4).

The most frequently reported countries were the United Kingdom (11%), Turkey (7%), Germany (5%), Italy (4%), France (4%), Greece (3%), Mexico (3%) and Spain (3%).

Fifty‐five percent (611) respondents from 79 countries described themselves as specialists in periodontology; 20% (*n* = 218) were working as part‐time or full‐time academics; 6% (*n* = 64) were working as dental hygienists or dental therapists and 12% (*n* = 128) as general dental practitioners; and 1% (*n* = 11) were undergraduate students in dentistry, dental hygiene or dental hygiene and therapy.

Ninety‐eight percent (*n* = 1094) respondents were aware of the new classification, and 78% (*n* = 869) reported that they use it; 14% (*n* = 155) used the classification ‘sometimes’ and 8% (*n* = 86) did not use it at all.

Seventy‐seven percent (*n* = 854) respondents reported that their national society had produced implementation guidance, 9% (*n* = 104) did not know and 13% (*n* = 149) stated that this was not the case. Those that did not use the 2018 Classification were almost all still using the 1999 Armitage Classification. When responding to the question: ‘What influences you to use the 2018 periodontal classification?’, the answers indicated that it was felt to be a ‘better’ more ‘sensitive’ and ‘precise’ classification. Others reported using it because they ‘had to’, and some used the new classification for ‘academic reasons’. Reasons for not using were that it was felt to be ‘too complicated’, whereas for some the insurance carriers did not yet accept the new classification, so using two systems was perceived as ‘inconvenient’.

Only 6% (*n* = 65) responded to ‘What could be changed to support you in using the new classification?’, with most of these responses asking for a simplification of the system. Ninety‐nine percent (*n* = 1104) rated ‘How easy is it to use the 2018 Classification?’ on a scale of 1 (not easy at all) to 5 (very easy), with a mean response of 3.4 (neither easy nor difficult), 555 (50%) rating its use as 4 or 5 (easy or very easy) and 22% (*n* = 241) rating it 2 or 1 (somewhat difficult or not easy at all).

Forty‐four percent (*n* = 482) respondents felt that the 2018 Classification met their needs, 43% (*n* = 474) reported that it ‘somewhat’ met their needs and 13% (*n* = 149) stated that it did not meet their needs. Fifty‐eight percent (*n* = 643) had not encountered problems when staging or grading with the new classification, whereas 42% (*n* = 462) had encountered problems. Of those who encountered problems, the aspects that they found difficult were a lack of previous examination data or reasons why teeth were previously lost; confusion with grade modifiers; confusion with complexity factors when staging; and difficulty determining stage III from stage IV.

The majority, 74% (*n* = 813), felt that the 2018 Classification was an improvement on the previous classification. Fifty‐right percent (*n* = 651) gave reasons why it was an improvement, such as a ‘clearer’ classification; the ‘inclusion of periodontal health’; ‘recognises the importance of bleeding on probing’; allows a ‘more individualised diagnosis’; the recognition of ‘patient susceptibility’; and its sensitivity to early disease. Twenty percent (*n* = 220) gave reasons why they felt it was not an improvement: that several felt the removal of aggressive periodontitis was not fully justifiable; that it is too time consuming for use in general practice; and it was considered by many to be overly complex to use. Finally, 61% (*n* = 677) responded to ‘What would you like to see in the 2018 Classification?’ and answers followed two themes. One theme focused on a desire for the classification to be simplified, and the second asked for reintroduction of aggressive periodontitis.

### 3.4 Discussion

This review was presented in two parts, both of which focused on the use of the 2018 Classification of Periodontal and Peri‐Implant Diseases and Conditions with regard to staging and grading periodontitis. Part 1 evaluated the performance metrics of the 2018 Classification, including diagnostic accuracy, prognostic performance and examiner reliability with descriptive statistics. This part of the review included 13 individual studies with moderate risk of bias. Part 2 of this review focused on barriers and facilitators to the implementation of the 2018 Classification, including 33 individual papers synthesised using thematic analysis, as well as 1113 responses to a web‐based survey. The results of Part 2 should be considered as having high risk of bias.

Eight studies assessed an element of diagnostic accuracy of the 2018 Classification, all using the 2012 AAP/CDC classification as the benchmark for comparison (Botelho et al. 2020; Brito et al. 2022; Costea et al. 2022; Li et al. 2022; Machado et al. 2020; Morales et al. 2022; Ortigara et al. 2021; Shi et al. 2024). The 2018 Classification was regarded as having high sensitivity for the detection of periodontitis, but the specificity was felt to be lower but acceptable. An explanation for these findings may be related to the change in case definition, whereby there is now a lower threshold for recognising loss of attachment as a disease process, that is, periodontitis. The clinical and radiographic progression from gingivitis to early periodontitis is subtle and this will inevitably impact on specificity measurements. However, many may consider the increased ability to detect early disease a strength and worth the slight loss in specificity. Prevention has been shown to be the most cost‐effective method of managing periodontitis (Economist Impact 2021), which focuses around risk factor modification and optimisation of self‐performed plaque biofilm control (Sanz, Herrera, et al. 2020) and can be performed by several members of the dental team, whereas more severe periodontitis stages require the input from special‐interest and specialist periodontal practitioners (NHS England 2022; Sanz, Herrera, et al. 2020). Recently, there have been assertions made in the literature that diagnosing more early cases may be detrimental to those with advanced disease due to spreading healthcare resources more thinly (Raittio et al. 2025). However, as noted above the management of these ‘grey’ area cases with early periodontitis is simple and cost effective and should have been offered to all gingivitis patients anyway. So, it seems unlikely that this will have an adverse impact on more advanced cases and, in the longer term, may reduce their prevalence. This view is supported by Part 2 of this review, where the emphasis of early detection of periodontitis was noted as enabling early active preventative strategies (Costea et al. 2022; Germen et al. 2021; Karaaslan et al. 2021; Miyamoto et al. 2019). Survey respondents also noted that the classification was ‘better’ more ‘sensitive’ and ‘precise’, which influenced them to use it in practice. The increased sensitivity was even felt to be an advantage when using partial‐mouth protocols in epidemiological studies (Botelho et al. 2020).

Three retrospective cohort studies assessed the prognostic capability of the 2018 Classification against the clinical endpoint of tooth loss (Dukka et al. 2022; El Sayed et al. 2022; Saleh et al. 2022). El Sayed et al. (2022) compared the 2018 Classification with the 1999 Armitage classification and found no difference between them for predicting tooth loss. Dukka et al. (2022) assessed the 2018 Classification against BSP‐I and reported that both models had excellent prognostic capability. Saleh et al. (2022) while comparing the staging and grading system with a number of Periodontal Risk Assessment Tools (PRATs) found that all performed well when considering Harrell's C index, with the PerioRisk tool performing the best. Several sources felt the staging and grading aspect of the classification was a facilitator to its implementation, with Schwendicke et al. (2020) noting that tooth loss was associated with the stage and grade, with more teeth lost in stage IV than III and with grade C than B (Aboalsaud et al. 2023; El Sayed et al. 2022; Miyamoto et al. 2019; Schwendicke et al. 2020). In addition, treatment costs showed a positive association with the grade (Schwendicke et al. 2020).

Three studies assessed elements of intra‐ and inter‐rater reliability related to the 2018 Classification (Marini et al. 2021; Abrahamian et al. 2022; Ravida et al. 2021). Inter‐rater reliability was found to be moderate (kappa value range 0.37–0.51) in two studies (Marini et al. 2021; Ravida et al. 2021) and intra‐rater reliability was moderate to very good (kappa 0.52–0.85) in one study (Abrahamian et al. 2022). In Part 2, barriers to implementing the 2018 Classification may partly explain this. For instance, Ertas et al. (2023) described the 2018 Classification as complex due to multiple clinical and radiographic factors, making staging and grading difficult. Gandhi et al. (2022) felt that there were multiple subjective factors, and Steigmann et al. (2021) felt that borderline cases required the use of ‘clinical judgement’ to arrive at an ‘appropriate diagnosis’. In the survey, themes also arose regarding complexity of the new classification such as subtle judgements or differences in interpretation, which could in part account for the slightly lower than ideal inter‐rater reliability. Ertas et al. (2023) suggested that a potential solution to this problem was the use of simple decision flowcharts to minimise confusion and inconsistent diagnoses.

Several clinical facilitators were noted regarding the implementation of the 2018 Classification. Multiple sources felt that the new classification promoted patient‐tailored diagnosis and treatment planning (Aboalsaud et al. 2023; Babay et al. 2019; Claydon et al. 2022; Karaaslan et al. 2021; Miyamoto et al. 2019; Schwendicke et al. 2020). It was noted that incorporating risk factors into the classification helped in providing individually tailored treatment plans (Babay et al. 2019) and in understanding an individual's likely response to treatment (Aboalsaud et al. 2023). Miyamoto et al. (2019) suggested that grading was crucial for planning preventive strategies and reinforcing the need for meticulous periodontal supportive therapy. A number of implementation strategies were deployed to help with the dissemination of the 2018 Classification, such as decision flowcharts, which aimed to simplify the process (Ertas et al. 2023; Parsegian et al. 2021; Sutthiboonyapan et al. 2020). Technological strategies were also used to automate/semi‐automate the classification process. A number of promising computational methods have been proposed, with one study finding that 80% of clinicians were positive about a digital tool to facilitate decision making (Ertas et al. 2023; Fidyawati et al. 2024; Guler Ayyildiz et al. 2024; Lee et al. 2022; Malmqvist et al. 2024; Meir et al. 2023; Patel et al. 2022; Sanchez‐Otalvaro et al. 2022). In addition, there were also regional implementation strategies with slight variations in their interpretations of the new classification. Notable instances were in the United Kingdom, United States, The Netherlands and Ireland (Jayawardena et al. 2021). The only local strategy that was described in detail was BSP‐I. Claydon et al. (2022) noted that it was possible to successfully implement BSP‐I in a general practice setting within a year of publication. Although BSP‐I is a reductionist interpretation of the 2018 periodontal classification, Dukka et al. (2022) showed that this had no effect on the classification allocation or the prognostic performance of the system.

A number of practical barriers were perceived regarding the implementation of the 2018 Classification. Some academics thought that the complexity of the system was challenging for students, which mirrored the practice setting (Gandhi et al. 2022). These views were shared by 8% of respondents in the survey who reported that they did not use the new classification often because it was ‘too complicated’. The strongest criticisms levelled at the 2018 Classification were in relation to barriers to its implementation for use in epidemiological studies. Concerns focused around the need for full‐mouth clinical and radiographic examination, which is not always possible or pragmatic in epidemiological studies, as well as the need to undertake a thorough history, for example, to assess reasons for tooth loss (Alawaji et al. 2022; Botelho et al. 2020; Costea et al. 2022; Du et al. 2023; Germen et al. 2021; Graham and Turner 2020; Holtfreter et al. 2024; Karaaslan et al. 2021; Ke et al. 2023; Malmqvist et al. 2024; Raittio et al. 2025; Schwendicke et al. 2020). However, it was accepted that the new classification was extremely comprehensive (Du et al. 2023). Further concerns were noted regarding the changing case definition, potentially leading to overestimation of disease prevalence (Raittio et al. 2025), although, as previously discussed, this is not necessarily a weakness and is paradigm dependent. It is important to recognise the difference between classification and diagnosis, as this may impact on the implementation of the 2018 Classification, especially in relation to epidemiological studies. Classification is the first step of arriving at a diagnosis, but diagnosis also contains information about current disease activity, for example, bleeding on probing and probing depth (Dietrich et al. 2019). Hence, a greater degree of clinical examination is required to arrive upon a diagnostic statement for each patient. This may be challenging to perform as part of larger epidemiological studies.

The survey reports from a variety of contexts internationally across a variety of professional groups, although, due to the survey being administered through the EFP, a bias is noted. That 98% of respondents are aware of the classification, and 77% of respondents report that their member society has produced local implementation guidance, should be regarded as a successful outcome. The survey results did reveal that, although 74% of respondents felt the 2018 Classification was an improvement on previous classifications, 66% felt it still did not meet their needs. It is encouraging that the classification provides an improved means of classifying disease for the practitioner but provides an insight that aligns with the barriers identified in Part 2 of this review, namely that there are potential avenues for adaptation to support the classification being used to its full potential. In free‐text results, the theme of ‘simplification’ was reported as a potential improvement to its current form. The results of the survey corroborate the findings from the reviews that the classification appears to be an improvement on previous classifications while noting some limitations to its ubiquitous uptake.

There are limitations that exist within this mixed‐methodology study. In Part 1, the theme of diagnostic accuracy was exceptionally heterogeneous, and this precluded undertaking a meta‐analysis. The study design under review was complicated due to the majority of assessments including the index test as the 2018 Classification, and evaluations were conducted with previous or adapted classifications, which limits its utility in being able to assess diagnostic accuracy. The majority of studies did not break their assessment of diagnostic accuracy between stage, grade, extent and composite diagnosis, and therefore a generalised view was provided, as shown in Appendix 7 of Supporting Information S1.

In Part 2, while it was encouraging that key themes were explored, there was a lack of primary qualitative data exploring the implementation in detail.

As the survey was distributed through the EFP MailChimp directory, which consists of any individual who has registered for an EFP event, it is possible that the majority of respondents are members of a periodontal society, which may introduce selection bias into the study. Potential generalisability is therefore limited and would be improved through adapted and additional sampling techniques. It must be accepted that a determined individual could submit deceptive responses to the survey, although the themes were so consistent that it is extremely unlikely that this would result in spurious results.

A deviation from the PROSPERO record was made with regard to risk of bias assessment in Part 2. There was no formal use of a tool such as the Mixed Methods Appraisal Tool (MMAT), which was due to the considerable variation in study type and outcomes collected that supported Part 2; in addition, the strength of evidence was not assessed using the GRADE approach as initially planned.

### 3.5 Conclusion

The 2018 Classification offers a novel means of classifying periodontal diseases. The results of this review provided some limited evidence that this classification is a potentially improved means of periodontal disease classification in comparison to previous systems. The evidence for diagnostic accuracy was limited due to the heterogeneity in index reference standards used in studies, but mild diagnostic concordance was seen when examining the composite periodontal diagnosis and previous classifications. The predictive ability of the classification was unclear regarding the endpoint of tooth loss, but it did show moderate examiner reliability. Further adaptation is required for the tool to be diagnostically accurate and reproducible and to offer predictive ability as its intended purpose.

Its implementation has potentially been limited by barriers such as its perceived complexity. However, facilitators such as local implementation strategies and adjunctive aids have supported practitioners in clinical settings. In addition, its promotion of patient‐tailored diagnostics and treatment planning was felt to be beneficial. However, its role in epidemiological studies would benefit from further refinements. With marginal adaptation, it is believed the 2018 Classification can be further enhanced to ensure that its utility is maximised as an internationally accepted classification tool.

If adaptation was considered, it would be valuable for future primary research to include robust diagnostic accuracy studies in a large sample size broadly representative of the population of interest prior to widespread implementation, with a clear differentiation between stage, grade, extent and composite diagnosis made. The authors would recommend further qualitative research exploring the challenges with implementing these changes across a variety of settings, including primary dental care.

## Author Contributions

**Nicola X. West:** conceptualization, methodology, supervision, project administration, writing – original draft, writing – review and editing. **Alexander Gormley:** methodology, investigation, formal analysis, writing – original draft, writing – review and editing. **Alexander J. Pollard:** methodology, investigation, formal analysis, writing – original draft, writing – review and editing. **Rossana Izzetti:** investigation, formal analysis, writing – review and editing. **Crystal Marruganti:** investigation, formal analysis, writing – review and editing. **Filippo Graziani:** conceptualization, methodology, supervision, project administration, writing – review and editing. All authors gave final approval and agreed to be accountable for all aspects of work ensuring integrity and accuracy.

## Ethics Statement

The authors have nothing to report.

## Conflicts of Interest

The authors declare no conflicts of interest.

## Supporting information


**Supporting Information S1.** Appendix 7: Part 1—Diagnostic accuracy summary table.
Appendix 8: Part 1—Prognostic efficacy summary table.
Appendix 9: Part 1—Examiner reliability summary table.

**Supporting Information S2.** Appendix 10: Part 1—Diagnostic accuracy quality appraisal.
Appendix 11: Part 1—Prognostic efficacy quality appraisal.
Appendix 12: Part 1—Examiner reliability quality appraisal.

**Supporting Information S3.** Appendix 13: Part 2—Code book.

**Supporting Information S4.** Appendix 14: Part 2—Survey.

**Supporting Information S5.** Appendix 15: Part 2—Data Extraction from Included studies with primary codes.

**Supporting Information S6.** Appendix 16: Part 2—Shortlisting full text.

## Data Availability

The data that support the findings of this study will be stored in the publicly available University of Bristol Research Data Repository once the paper has been published. Data will be made available to researchers subject to the agreement of the University of Bristol Data Access Committee.

## Appendix 1 Part 1—Search Strategy

*OvidSP versions of Medline and EMBASE* (inception—21 June 2024)

**Table:**

1.	exp “PERIODONTICS”/
2.	(periodontal* or periodontic* or periodontit*).mp. [mp=title, book title, abstract, original title, name of substance word, subject heading word, floating sub‐heading word, keyword heading word, organism supplementary concept word, protocol supplementary concept word, rare disease supplementary concept word, unique identifier, synonyms, population supplementary concept word, anatomy supplementary concept word]
3.	1 or 2
4.	(classifi* or diagnos* or EFP or “European Federation Periodontology” or AAP or “American Association Periodontology” or workshop or new or updated or “2017” or “2018”).mp. [mp=title, book title, abstract, original title, name of substance word, subject heading word, floating sub‐heading word, keyword heading word, organism supplementary concept word, protocol supplementary concept word, rare disease supplementary concept word, unique identifier, synonyms, population supplementary concept word, anatomy supplementary concept word]
5.	3 and 4
6.	exp “Sensitivity and Specificity”/
7.	(sensitive or sensitivity or specific or specificity or predict* or reference value or reference standard or ROC or estimate* or constructiv* or prognos* or reproduce or reproducibility or perform*).mp. [mp=title, book title, abstract, original title, name of substance word, subject heading word, floating sub‐heading word, keyword heading word, organism supplementary concept word, protocol supplementary concept word, rare disease supplementary concept word, unique identifier, synonyms, population supplementary concept word, anatomy supplementary concept word]
8.	6 or 7
9.	5 and 8
10.	limit 9 to yr=“2018–Current”

## Appendix 2 Part 2—Search Strategy

*OvidSP versions of Medline and EMBASE* (inception—21 June 2024)

**Table:**

1.	exp “PERIODONTICS”/
2.	(periodontal* or periodontic* or periodontit*).mp. [mp=title, book title, abstract, original title, name of substance word, subject heading word, floating sub‐heading word, keyword heading word, organism supplementary concept word, protocol supplementary concept word, rare disease supplementary concept word, unique identifier, synonyms, population supplementary concept word, anatomy supplementary concept word]
3.	1 or 2
4.	(classifi* or diagnos* or EFP or “European Federation Periodontology” or AAP or “American Association Periodontology” or workshop or new or updated or “2017” or “2018”).mp. [mp=title, book title, abstract, original title, name of substance word, subject heading word, floating sub‐heading word, keyword heading word, organism supplementary concept word, protocol supplementary concept word, rare disease supplementary concept word, unique identifier, synonyms, population supplementary concept word, anatomy supplementary concept word]
5.	3 and 4
10.	limit 9 to yr=“2018–Current”

## Appendix 3 Part 1—Papers Excluded From Full‐Text Screening

**Table:**

Reference	Reason
Abbas, Y., Elsaadany, B. & Ghallab, N. 2023. Prevalence of different stages of periodontal diseases among a sample of young adult obese Egyptian patients: A hospital based Cross‐sectional study over 1 year. BMC oral health, 23, 573.	Does not provide assessment between 2018 Classification and other classification
Aboalsaud, K. M., Foster, N. L., Yu, S.‐H., Sweier, D. G. & Rulli, D. 2023. Periodontal staging and grading: An international dental hygiene education survey. International journal of dental hygiene, 21, 283–290.	Does not provide assessment between 2018 Classification and other classification
Delatola, C., Loos, B. G. & Laine, M. L. 2020. Three periodontitis phenotypes: Bone loss patterns, antibiotic‐surgical treatment and the new classification. Journal of clinical periodontology, 47, 1371–1378.	Does not provide assessment between 2018 Classification and other classification
Deng, K., Pelekos, G., Jin, L. & Tonetti, M. S. 2021. Diagnostic accuracy of self‐reported measures of periodontal disease: A clinical validation study using the 2017 case definitions. Journal of clinical periodontology, 48, 1037–1050.	Outcomes relate to the self‐reported questions and not classification
Destaffany, A. M., Gurenlian, J. a. R. & Bono, L. K. 2023. Investigating periodontal diagnosis and treatment at one dental school. European Journal of Dental Education: Official Journal of the Association for Dental Education in Europe, 27, 535–540.	Does not provide assessment between 2018 Classification and other classification
Du, M., Mo, Y., Li, A., Ge, S. & Peres, M. A. 2023. Assessing the surveillance use of 2018 EFP/AAP classification of periodontitis: A validation study and clustering analysis. Journal of periodontology, 94, 1254–1265.	Does not include primary outcome
Ghassib, I. H., Batarseh, F. A., Wang, H. L. & Borgnakke, W. S. 2021. Clustering by periodontitis‐associated factors: A novel application to NHANES data. Journal of Periodontology, 92, 1136–1150.	Does not include primary outcome
Gheorghe, D. N., Nicolae, F. M., Popescu, D. M., Ciobanu, S. & Surlin, P. 2024. Clinical and Demographic Profiling of Periodontal Diseases: A Retrospective Analysis Using the 2018 Periodontal Classification Algorithm. Current health sciences journal, 50, 29–35. Abdelrasoul, M. & Fouad, A. (2022) Calibration of periodontal diagnosis based on the 2017 classification of periodontal diseases among dental interns at a private dental school. Journal of Clinical Periodontology, 49, pp. 67.	Does not provide assessment between 2018 Classification and other classification
Gurbuz, S. & Altikat, M. 2023. The association between periodontitis patients' chief complaints and the stage of periodontitis: A clinical retrospective study. Clinical oral investigations, 27, 6261–6272.	Does not provide assessment between 2018 Classification and other classification
Ju, X., Harford, J., Luzzi, L., Mejia, G. & Jamieson, L. M. 2022. A Longitudinal Study of Chronic Periodontitis in Two Cohorts of Community‐Dwelling Elderly Australians. International journal of environmental research and public health, 19.	Does not include primary outcome
Ke, L., Nogueira, G. & Thomson, W. M. 2023. Influence of case definitions on epidemiological estimates of periodontitis prevalence and its associations with smoking and OHRQoL. Community dentistry and oral epidemiology, 51, 194–200.	Does not include primary outcome
Marini, L., Tomasi, C., Gianserra, R., Graziani, F., I, L., Merli, M., Nibali, L., Roccuzzo, M., Sforza, N. M., Tonetti, M. S., Deli, F., Papi, P., Murro, B. D., Rojas, M. A. & Pilloni, A. 2023. Reliability assessment of the 2018 classification case definitions of peri‐implant health, peri‐implant mucositis, and peri‐implantitis. Journal of periodontology, 94, 1461–1474.	Not related to the classification of periodontal disease
Matijevic, N., Stipic, M. & Music, L. 2019. Is the 2017 classification of periodontal diseases clinician‐friendly? review of the classification through case analysis. Acta Stomatologica Croatica, 53, 188.	Does not provide assessment between 2018 Classification and other classification
Nascimento, G. G., Dahlen, G., Lopez, R. & Baelum, V. 2020. Periodontitis phenotypes and clinical response patterns to non‐surgical periodontal therapy: Reflections on the new periodontitis classification. European journal of oral sciences, 128, 55–65.	Does not provide assessment between 2018 Classification and other classification
Parsegian, K., Ayilavarapu, S., Patel, T., Henson, H. A. & Angelov, N. 2021. Flowcharts improve periodontal diagnosis by dental and dental hygiene students. Canadian journal of dental hygiene: CJDH = Journal canadien de l'hygiene dentaire: JCHD, 55, 137–147.	Does not provide assessment between 2018 Classification and other classification
Reiniger, A. P. P., Londero, A., Barrachini, A., Ferreira, T. D. G. M., Da Rocha, J. M., Moreira, C. H. C. & Kantorski, K. Z. 2020. Validity of self‐reported measures for periodontitis surveillance in a rural sample. Journal of periodontology, 91, 617–627.	Outcomes relate to the self‐reported questionnaire not the classification
Raittio, E. & Baelum, V. 2023. Justification for the 2017 periodontitis classification in the light of the Checklist for Modifying Disease Definitions: A narrative review. Community Dentistry and Oral Epidemiology, 51, 1169–1179.	Does not provide assessment between 2018 Classification and other classification
Sutthiboonyapan, P., Wang, H.‐L. & Charatkulangkun, O. 2020. Flowcharts for Easy Periodontal Diagnosis Based on the 2018 New Periodontal Classification. Clinical advances in periodontics, 10, 155–160.	Does not provide assessment between 2018 Classification and other classification
Tokede, B., Br, On, R., Lee, C.‐T., Lin, G.‐H., White, J., Yansane, A., Jiang, X., Kalenderian, E. & Walji, M. 2024. Development and validation of a rule‐based algorithm to identify periodontal diagnosis using structured electronic health record data. Journal of clinical periodontology, 51, 547–557.	Does not provide assessment between 2018 Classification and other classification

## Appendix 4 Part 2—Papers Excluded From Full‐Text Screening

**Table:**

Reference	Reason
Abdelrasoul, M. & Fouad, A. (2022) ‘Calibration of periodontal diagnosis based on the 2017 classification of periodontal diseases among dental interns at a private dental school’. Journal of Clinical Periodontology, 49, pp. 67.	No relevant data
Abou‐Arraj, R. V., Kaur, M., Alkhoury, S., Swain, T. A., Geurs, N. C. & Souccar, N. M. (2021) ‘The new periodontal disease classification: Level of agreement on diagnoses and treatment planning at various dental education levels’. Journal of Dental Education, 85 (10), pp. 1627–1639.	No relevant data
Ahmedbeyli, C. R., Mammadov, F. Y., Garayev, R. M., Mammadov, A. M. & Guliyeva, V. E. (2020) ‘New international classification of periodontal and peri‐implant diseases’. Azerbaijan Medical Journal, 2020 (2), pp. 106–110.	Not available in English
Akinci, S., Gulsahi, A. & Alaaddinoglu, E. E. (2022) ‘How does the periodontitis stage and grade influence extraction decision for dental implant placement?’. Journal of Clinical Periodontology, 49, pp. 67.	Paper not retrieved
Almutairi, A. S. (2022) ‘Assessment of general dentists' knowledge of the new classification of periodontal and peri‐implant diseases and conditions 2017: A cross‐sectional study in Saudi Arabia’. Pakistan Journal of Medical and Health Sciences, 16 (2), pp. 540–544.	No relevant data
Anonymous (2018) ‘New classification of periodontal and peri‐implant diseases and conditions’. British Dental Journal, 225 (1), pp. 5.	Paper not retrieved
Brito, L. F., Taboza, Z. A., Silveira, V. R., Teixeira, A. K. & Rego, R. O. (2022) ‘Diagnostic accuracy of severe periodontitis case definitions: Comparison of the CDC/AAP, EFP/AAP, and CPI criteria’. Journal of Periodontology, 93 (6), pp. 867–876.	No relevant data
Chang, H.‐J., Lee, S.‐J., Yong, T.‐H., Shin, N.‐Y., Jang, B.‐G., Kim, J.‐E., Huh, K.‐H., Lee, S.‐S., Heo, M.‐S., Choi, S.‐C., Kim, T.‐I. & Yi, W.‐J. (2020) ‘Deep Learning Hybrid Method to Automatically Diagnose Periodontal Bone Loss and Stage Periodontitis’. Scientific Reports, 10 (1), pp. 7531.	No relevant data
Chitsazi, M., Babaloo, A. & Mohammadi, H. (2021) ‘Questions on the new classification of periodontal and preimplantation diseases’. Journal of Advanced Periodontology & Implant Dentistry, 13 (2), pp. 95–96.	No relevant data
Ciurea, A., Stanomir, A., Surlin, P., Micu, I. C., Pamfil, C., Leucuta, D. C., Rednic, S., Rasperini, G., Soanca, A., Tigu, A. B., Roman, A., ra, Picos, A. & Delean, A. G. (2024) ‘Insights into the relationship between periodontitis and systemic sclerosis based on the new periodontitis classification (2018): A cross‐sectional study’. Diagnostics (Basel, Switzerland), 14 (5)	No relevant data
Coachman, C., Blatz, M. B., Bohner, L. & Sesma, N. (2021) ‘Dental software classification and dento‐facial interdisciplinary planning platform’. Journal of Esthetic and Restorative Dentistry: Official Publication of the American Academy of Esthetic Dentistry … [et al.], 33 (1), pp. 99–106.	No relevant data
Costa, R. P., Resende, M. S., Pinto, M. G. & Mendes, L. (2019) ‘Periodontal diagnosis: A decision flowchart for the new classification’. Revista Portuguesa de Estomatologia, Medicina Dentaria e Cirurgia Maxilofacial, 60 (4), pp. 189–196.	Not available in English
Crome, M., Adam, K., Flohr, M., Rahman, A., er & Staufenbiel, I. (2021) ‘Application of the inverted classroom model in the teaching module “new classification of periodontal and peri‐implant diseases and conditions” during the COVID‐19 pandemic’. GMS Journal for Medical Education, 38 (5), pp. Doc89.	No relevant data
Cuenin, M. F. & Rasheed, A. (2020) ‘Proceedings from the 2017 world workshop on the classification of periodontal and peri‐implant diseases and conditions – Integration in the pre‐doctoral clinic setting’. Journal of Dental Education.	No relevant data
De Almeida, S. D., Richter, G. M., de Coo, A., Jepsen, S., Kapferer‐Seebacher, I., Dommisch, H., Berger, K., Laudes, M., Lieb, W., Loos, B. G., van der Velde, N., van Schoor, N., de Groot, L., Blanco, J., Carracedo, A., Cruz, R. & Schaefer, A. S. (2024) ‘A genome‐wide association study meta‐analysis in a European sample of stage III/IV grade C periodontitis patients <=35 years of age identifies new risk loci’. Journal of Clinical Periodontology, 51 (4), pp. 431–440.	No relevant data
Deng, K., Pelekos, G., Jin, L. & Tonetti, M. S. (2021) ‘Diagnostic accuracy of self‐reported measures of periodontal disease: A clinical validation study using the 2017 case definitions’. Journal of Clinical Periodontology, 48 (8), pp. 1037–1050.	No relevant data
Dietrich, T., Ower, P., Tank, M., West, N. X., Walter, C., Needleman, I., Hughes, F. J., Wadia, R., Milward, M. R., Hodge, P. J. & Chapple, I. L. C. (2019) ‘Periodontal diagnosis in the context of the 2017 classification system of periodontal diseases and conditions – Implementation in clinical practice’. British Dental Journal, 226 (1), pp. 16–22.	No relevant data
Dorri, M. (2018) ‘Periodontal diseases: New classification for periodontal diseases’. British Dental Journal, 225 (8), pp. 686.	No relevant data
Feng, A.‐C., Tsai, S.‐C., Lin, Y.‐P., Tsai, K.‐Z. & Lin, G.‐M. (2022) ‘Erythrocyte indices and localized stage II/III periodontitis in military young men and women: CHIEF oral health study’. BMC Oral Health, 22 (1), pp. 404.	No relevant data
Forrest, J. L. & Lavigne, S. E. (2018) ‘What does research tell us about the future of dental hygiene? A New Classification of Periodontal Diseases: A paradigm shift for all!’. Journal of Dental Hygiene: JDH, 92 (4), pp. 4–5.	No relevant data
Garyga, V., Pochelu, F., Thivichon‐Prince, B., Aouini, W., Santamaria, J., Lambert, F., Maucort‐Boulch, D., Gueyffier, F., Gritsch, K. & Grosgogeat, B. (2019) ‘GoPerio – Impact of a personalized video and an automated two‐way text‐messaging system in oral hygiene motivation: study protocol for a randomized controlled trial’. Trials, 20 (1), pp. 699.	No relevant data
Gheorghe, D. N., Nicolae, F. M., Popescu, D. M., Ciobanu, S. & Surlin, P. (2024) ‘Clinical and demographic profiling of periodontal diseases: A retrospective analysis using the 2018 periodontal classification algorithm’. Current Health Sciences Journal, 50 (1), pp. 29–35.	No relevant data
Graetz, C., Mann, L., Krois, J., Salzer, S., Kahl, M., Springer, C. & Schwendicke, F. (2019) ‘Comparison of periodontitis patients' classification in the 2018 versus 1999 classification’. Journal of Clinical Periodontology, 46 (9), pp. 908–917.	No relevant data
Gul, S. N. S., Eminoglu, D. O., Laloglu, E., Aydin, T. & Dilsiz, A. (2024) ‘Salivary and serum asprosin hormone levels in the 2018 EFP/AAP classification of periodontitis stages and body mass index status: A case‐control study’. Clinical Oral Investigations, 28 (1), pp. 91.	No relevant data
Gurbuz, S. & Altikat, M. (2023) ‘The association between periodontitis patients' chief complaints and the stage of periodontitis: A clinical retrospective study’. Clinical Oral Investigations, 27 (10), pp. 6261–6272.	No relevant data
Hadler‐Olsen, E., Petrenya, N., Jonsson, B., Steingrimsdottir, O. A., Stubhaug, A. & Nielsen, C. S. (2024) ‘Periodontitis is associated with decreased experimental pressure pain tolerance: The Tromso Study 2015–2016’. Journal of Clinical Periodontology, 51 (7), pp. 874–883.	No relevant data
Hamdan, N., Al‐Waeli, H., Tamimi, F. & Berrizbeitia, F. (2022) ‘Development and validation of an online tool for the diagnosis of periodontal diseases based on 2018 classification of periodontal and peri‐implant diseases and conditions’. Journal of Clinical Periodontology, 49, pp. 166.	No relevant data
Harcke, K., Lindunger, A., Kollinius, E., Gebreslassie, M., Ugarph Morawski, A., Nylen, C., Peterson, M., Yucel‐Lindberg, T., Ostenson, C.‐G., Skott, P. & Saleh Stattin, N. (2024) ‘Observational study of selective screening for prediabetes and diabetes in a real‐world setting: An interprofessional collaboration method between public dental services and primary health care in Sweden’. Scandinavian Journal of Primary Health Care, 42 (1), pp. 170–177.	No relevant data
Harrel, S. K., Cobb, C. M., Sottosanti, J. S., Sheldon, L. N. & Rethman, M. P. (2022) ‘Clinical Decisions Based on the 2018 Classification of Periodontal Diseases’. Compendium of Continuing Education in Dentistry (Jamesburg, N.J.: 1995), 43 (1), pp. 52–56.	No relevant data
Hellyer, P. (2021) ‘Teaching the new periodontal disease classification’. British Dental Journal, 230 (11), pp. 752.	No relevant data
Herrera, D., Berglundh, T., Schwarz, F., Chapple, I., Jepsen, S., Sculean, A., Kebschull, M., Papapanou, P. N., Tonetti, M. S., Sanz, M., Aimetti, M., Blanco, J., Bostanci, N., Bouchard, P., Bozic, D., Buduneli, N., Calciolari, E., Carra, M. C., Cosgarea, R., Cosyn, J., Dannewitz, B., Danser, M., de Tapia, B., de Waal, Y., Derks, J., Dommisch, H., Donos, N., Eaton, K., Eickholz, P., Kuru, B. E., Figuero, E., Goldstein, M., Graziani, F., Grischke, J., Guerra, F., Heitz‐Mayfield, L., Jepsen, K., Kirkevang, L. L., Koldsl, O. C., Lambert, F., Linares, A., Loos, B., Madianos, P., Matesanz, P., Melo, P., Molina, A., Corti, V. M., Montero, E., Muller, F., Nibali, L., Pascual, A., Polyzois, I., Quirynen, M., Ramanauskaite, A., Rederiene, G., Renvert, S., Roccuzzo, M., Sahrmann, P., Salvi, G., Sanchez, N., Sanz‐Sanchez, I., Shapira, L., Stavropoulos, A., Stiesch, M., Tamosiunaite, I., Teughels, W., Tomasi, C., Trombelli, L., Vassilopoulos, S., Verket, A., West, N. & Wilensky, A. (2023) ‘Prevention and treatment of peri‐implant diseases‐The EFP S3 level clinical practice guideline’. Journal of Clinical Periodontology, 50, pp. 4–76.	No relevant data
Huynh, A. V., Latimer, J. M., Daubert, D. M. & Roberts, F. A. (2022) ‘Integration of a new classification scheme for periodontal and peri‐implant diseases through blended learning’. Journal of Dental Education, 86 (1), pp. 51–56.	No relevant data
Ibraheem, W. I., Bhati, A. K., Essa Ageeli, F. M., Sufyani, R. A., Ahmed Darraj, M., Ageeli, E. O., Mobarki, K. M., Alhazmi, M. Y. & Mohamed Beshir, S. E. (2024) ‘Association between asthma and periodontitis’. Bioinformation, 20 (1), pp. 59–64.	No relevant data
Immich, F., Cotti, E., Pirani, C. & Rossi‐Fedele, G. (2024) ‘What is new in the 2023 European Society of Endodontology S3‐level clinical practice guidelines?’. International Endodontic Journal.	No relevant data
Jokstad, A. (2019) ‘The 2018 AAP/EFP classification of periodontal diseases, a focus on “risks” as a faux ami and language gone on holiday’. Clinical and experimental dental research, 5 (5), pp. 449–451.	No relevant data
Kakar, A., Blanchard, S., Shin, D., Maupome, G., Eckert, G. J. & John, V. (2022) ‘Periodontal diagnosis and treatment planning – An assessment of the understanding of the new classification system’. Journal of Dental Education, 86 (12), pp. 1573–1580.	No relevant data
Kim, H.‐D., Lee, C.‐S., Cho, H.‐J., Jeon, S., Choi, Y.‐N., Kim, S., Kim, D., Jin Lee, H., Vu, H., Jeong, H.‐J. & Kim, B. (2020) ‘Diagnostic ability of salivary matrix metalloproteinase‐9 lateral flow test point‐of‐care test for periodontitis’. Journal of Clinical Periodontology, 47 (11), pp. 1354–1361.	No relevant data
Klinger, A. (2022) ‘Periodontal classification revisited: The classification system in the test of time’. Compendium of Continuing Education in Dentistry (Jamesburg, N.J.: 1995), 43 (3), pp. 184–188.	No relevant data
Kornman, K. S. & Papapanou, P. N. (2020) ‘Clinical application of the new classification of periodontal diseases: Ground rules, clarifications and “gray zones”’. Journal of Periodontology, 91 (3), pp. 352–360.	No relevant data
Lee, Y.‐H., Hong, S.‐J., Lee, G.‐J., Shin, S.‐I., Hong, J.‐Y., Chung, S. W. & Lee, Y.‐A. (2024) ‘Investigation of periodontitis, halitosis, xerostomia, and serological characteristics of patients with osteoarthritis and rheumatoid arthritis and identification of new biomarkers’. Scientific Reports, 14 (1), pp. 4316.	No relevant data
Li, H.‐J., Zhao, D., Xu, X., Yu, R., Zhang, F., Cheng, T., Zheng, Z., Yang, H., Yang, C., Yao, J., Wen, P. & Jin, L. (2022) ‘Diagnostic performance of the AAP/EFP classification and the CDC/AAP case definition among pregnant women and a practical screening tool for maternal periodontal diseases’. Journal of Periodontal Research, 57 (5), pp. 960–968.	No relevant data
Matijevic, N., Stipic, M. & Music, L. (2019) ‘Is the 2017 classification of periodontal diseases clinician‐friendly? review of the classification through case analysis’. Acta Stomatologica Croatica, 53 (2), pp. 188.	No relevant data
Meir, H., Miller, H., Saminsky, M., Levin, L. & Slutzkey, G. (2022) ‘A digital support system for the new 2017 world workshop periodontal classification – A pilot validation study of the “PerioClassApp”’. Journal of Clinical Periodontology, 49, pp. 58–59.	No relevant data
Meng, H. X. (2019) ‘[2018 world new classification of periodontal and peri‐implant diseases and conditions]’. Zhonghua kou qiang yi xue za zhi = Zhonghua kouqiang yixue zazhi = Chinese Journal of Stomatology, 54 (2), pp. 73–78.	Paper not retrieved
Montero, E., Herrera, D., Sanz, M., Dhir, S., Van Dyke, T. & Sima, C. (2019) ‘Development and validation of a predictive model for periodontitis using NHANES 2011–2012 data’. Journal of Clinical Periodontology, 46 (4), pp. 420–429.	Paper not retrieved
Morales, A., Munoz, G., Corral, C., Espinoza, I., Fuentes, A. D., Cavalla, F., Baeza, M., Jara, G., Giacaman, R. A., Suazo, C., Bevensee, I. & Gamonal, J. (2022) ‘Developing a protocol for a preventive oral health exam for elderly people (EDePAM) using E‐Delphi methodology’. Brazilian Oral Research, 36, pp. e013.	No relevant data
Morales, A., Strauss, F. J., Hammerle, C. H. F., Rom, ini, M., Cavalla, F., Baeza, M., Sanz, M. & Gamonal, J. (2022) ‘Performance of the 2017 AAP/EFP case definition compared with the CDC/AAP definition in population‐based studies’. Journal of Periodontology, 93 (7), pp. 1003–1013.	No relevant data
Nis, D., Struillo, X., Vincent‐Bugnas, S., Range, H. & Gosset, M. (2019) ‘A new classification of periodontal diseases’. Actualites Pharmaceutiques, 58 (589), pp. 49–52.	Paper not retrieved
Nisanci Yilmaz, M. N., Bulut, S. & Bakirarar, B. (2022) ‘Impact of stage‐grade of periodontitis and self‐reported symptoms on oral health‐related quality of life’. International Journal of Dental Hygiene, 20 (2), pp. 291–300.	No relevant data
Novello, S., Ferri, L., Brezulier, D. & Jeanne, S. (2020) ‘[A new classification in Periodontology (Chicago 2017). Impact in Orthodontics]’. Une nouvelle classification en parodontologie (Chicago 2017). Impact en orthodontie, 91 (1), pp. 35–40.	Not available in English
Oh, S. L., Yang, J. S. & Kim, Y. J. (2021) ‘Discrepancies in periodontal classification among dentists with different educational backgrounds’. Journal of Dental Education, 85 (2), pp. 252.	No relevant data
Oh, S.‐L., Yang, J. S. & Kim, Y. J. (2021) ‘Discrepancies in periodontitis classification among dental practitioners with different educational backgrounds’. BMC Oral Health, 21 (1), pp. 39.	Paper not retrieved
Orgeas, G. V., Nastri, L., Ausenda, F., Guglielmi, D., Audagna, M. & Ferrarotti, F. (2022) ‘Periodontal diagnosis and AAP‐EFP 2017 CLASSIFICATION’. Dental Cadmos, 90 (1), pp. 3–20.	Paper not retrieved
Ortigara, G. B., Mario Ferreira, T. d. G., Tatsch, K. F., Romito, G. A., re, Ardenghi, T. M., Sfreddo, C. S. & Moreira, C. H. C. (2021) ‘The 2018 EFP/AAP periodontitis case classification demonstrates high agreement with the 2012 CDC/AAP criteria’. Journal of Clinical Periodontology, 48 (7), pp. 886–895.	No relevant data
Pakdeesettakul, S., Charatkulangkun, O., Lertpimonchai, A., Wang, H.‐L. & Sutthiboonyapan, P. (2022) ‘Simple flowcharts for periodontal diagnosis based on the 2018 new periodontal classification increased accuracy and clinician confidence in making a periodontal diagnosis: A randomized crossover trial’. Clinical Oral Investigations, 26 (12), pp. 7021–7031.	No relevant data
Pan, L. F. & Shi, D. (2024) ‘[Predictive value of tooth loss risk with 2018 new classification of periodontitis: A literature review]’. Zhonghua kou qiang yi xue za zhi = Zhonghua kouqiang yixue zazhi = Chinese Journal of Stomatology, 59 (6), pp. 622–626.	Not available in English
Pini Prato, G. & Di Gianfilippo, R. (2021) ‘On the value of the 2017 classification of phenotype and gingival recessions’. Journal of Periodontology, 92 (5), pp. 613–618.	No relevant data
Raittio, E. & Baelum, V. (2023) ‘Justification for the 2017 periodontitis classification in the light of the Checklist for Modifying Disease Definitions: A narrative review’. Community Dentistry and Oral Epidemiology, 51 (6), pp. 1169–1179.	No relevant data
Ramya, V., Hussain, S. T., Padmanabhan, P. & Harshitha, R. T. (2019) ‘Personalised periodontal medicine‐a paradigm shift’. Indian Journal of Public Health Research and Development, 10 (11), pp. 3274–3277.	No relevant data
Ravida, A., Qazi, M., Troiano, G., Saleh, M. H. A., Greenwell, H., Kornman, K. & Wang, H.‐L. (2020) ‘Using periodontal staging and grading system as a prognostic factor for future tooth loss: A long‐term retrospective study’. Journal of Periodontology, 91 (4), pp. 454–461.	No relevant data
Ravida, A., Rodriguez, M. V., Saleh, M. H. A., Galli, M., Qazi, M., Troiano, G., Wang, H.‐L. & Moreno, P. G. (2021) ‘The correlation between history of periodontitis according to staging and grading and the prevalence/severity of peri‐implantitis in patients enrolled in maintenance therapy’. Journal of Periodontology, 92 (11), pp. 1522–1535.	No relevant data
Ravida, A., Travan, S., Saleh, M. H. A., Greenwell, H., Papapanou, P. N., Sanz, M., Tonetti, M., Wang, H.‐L. & Kornman, K. (2021) ‘Agreement among international periodontal experts using the 2017 World Workshop classification of periodontitis’. Journal of Periodontology, 92 (12), pp. 1675–1686.	No relevant data
Ravida, A., Troiano, G., Qazi, M., Saleh, M. H. A., Lo Russo, L., Greenwell, H., Giannobile, W. V. & Wang, H.‐L. (2020) ‘Development of a nomogram for the prediction of periodontal tooth loss using the staging and grading system: A long‐term cohort study’. Journal of Clinical Periodontology, 47 (11), pp. 1362–1370.	No relevant data
Raza, M., Abud, D. G., Wang, J. & Shariff, J. A. (2024) ‘Ease and practicability of the 2017 classification of periodontal diseases and conditions: A study of dental electronic health records’. BMC Oral Health, 24 (1), pp. 621.	No relevant data
Relvas, M., Lopez‐Jarana, P., Monteiro, L., Pacheco, J. J., Braga, A. C. & Salazar, F. (2022) ‘Study of Prevalence, Severity and Risk Factors of Periodontal Disease in a Portuguese Population’. Journal of Clinical Medicine, 11 (13).	No relevant data
Romano, F., Perotto, S., Castiglione, A. & Aimetti, M. (2019) ‘Prevalence of periodontitis: Misclassification, under‐recognition or over‐diagnosis using partial and full‐mouth periodontal examination protocols’. Acta Odontologica Scandinavica, 77 (3), pp. 189–196.	No relevant data
Ruetters, M., Gehrig, H., Kronsteiner, D., Schuessler, D. L. & Kim, T.‐S. (2022) ‘Prevalence of endo‐perio lesions according to the 2017 World Workshop on the Classification of Periodontal and Peri‐Implant Disease in a university hospital’. Quintessence International (Berlin, Germany: 1985), 53 (2), pp. 134–142.	No relevant data
Sanz, M., Papapanou, P. N., Tonetti, M. S., Greenwell, H. & Kornman, K. (2020) ‘Guest editorial: Clarifications on the use of the new classification of periodontitis’. Journal of Clinical Periodontology, 47 (6), pp. 658–659.	No relevant data
Sasaki, T., Otsuka, K., Yoshikawa, Y., Omagari, K., Hashimoto, T., Suzuki, K. & Tamura, A. (2023) ‘Tooth Loss as a Predictor of Postoperative Complications and Prognosis in Patients with Colorectal Cancer’. Journal of Gastrointestinal Cancer, 54 (4), pp. 1261–1267.	No relevant data
Shao, J. L., Yu, Y., Lyu, C. X. & Ge, S. H. (2022) ‘[Introduction and interpretation of the European Federation of Periodontology S3 level clinical practice guideline for treatment of periodontitis]’. Zhonghua kou qiang yi xue za zhi = Zhonghua kouqiang yixue zazhi = Chinese journal of stomatology, 57 (12), pp. 1202–1208.	No relevant data
Shi, J., Zhou, N., He, B., Hong, X., Guo, W., Jiang, L., Wang, C., Lei, L. & Li, H. (2023) ‘Diagnostic accuracy of severe periodontitis for Ramfjord teeth based on different classifications’. Oral Diseases.	Paper not retrieved
Shimchuk, A. A., Weinstein, B. F. & Daubert, D. M. (2021) ‘The impact of a change in classification criteria on the prevalence of peri‐implantitis: A cross‐sectional analysis’. Journal of Periodontology, 92 (9), pp. 1339–1346.	No relevant data
Siqueira, R., Andrade, N., Yu, S.‐H., Kornman, K. S. & Wang, H.‐L. (2021) ‘Challenges and Decision Making for the Classification of Two Complex Periodontal Cases’. Clinical Advances in Periodontics, 11 (2), pp. 103–110.	No relevant data
Smitha, K., Maria Thomas, J., Shankareswari, T. R., Affsha, S. & Anvitha, D. (2020) ‘Critical appraisal of the classification of periodontal and peri‐implant diseases and conditions (2017)’. Oral Diseases, 26 (5), pp. 1090–1091.	No relevant data
Stodle, I. H., Sen, A., Hovik, H., Verket, A., Koldsl & O. C. (2023) ‘Association between periodontitis stages and self‐reported diseases in a Norwegian population: The HUNT study’. BMC Oral Health, 23 (1), pp. 999.	Paper not retrieved
Stodle, I. H., Verket, A., Hovik, H., Sen, A., Koldsl & O. C. (2021) ‘Prevalence of periodontitis based on the 2017 classification in a Norwegian population: The HUNT study’. Journal of Clinical Periodontology, 48 (9), pp. 1189–1199.	Paper not retrieved
Takedachi, M., Shimabukuro, Y., Sawada, K., Koshimizu, M., Shinada, K., Asai, H., Mizoguchi, A., Hayashi, Y., Tsukamoto, A., Miyago, M., Nishihara, F., Nishihata, T., Shimabukuro, M., Kurakami, H., Sato, T., Hamazaki, Y., Iwayama, T., Fujihara, C. & Murakami, S. (2022) ‘Evaluation of periodontitis‐related tooth loss according to the new 2018 classification of periodontitis’. Scientific Reports, 12 (1), pp. 11893.	No relevant data
Tonetti, M. S. & Sanz, M. (2019) ‘Implementation of the new classification of periodontal diseases: Decision‐making algorithms for clinical practice and education’. Journal of Clinical Periodontology, 46 (4), pp. 398–405.	No relevant data
Trindade, D., Carvalho, R., Machado, V., Chambrone, L., ro, Mendes, J. J. & Botelho, J. (2023) ‘Prevalence of periodontitis in dentate people between 2011 and 2020: A systematic review and meta‐analysis of epidemiological studies’. Journal of Clinical Periodontology, 50 (5), pp. 604–626.	No relevant data
Varela‐Lopez, A., Bullon, B., Gallardo, I., Quiles, J. L. & Bullon, P. (2024) ‘Association of specific nutritional intake with periodontitis’. BMC Oral Health, 24 (1), pp. 640.	No relevant data
Velosa‐Porras, J. & Rodriguez Malagon, N. (2024) ‘Prevalence and social determinants of periodontal disease in Colombian pregnant women’. Community Dentistry and Oral Epidemiology, 52 (2), pp. 207–216.	No relevant data
Verhulst, M. J. L., Teeuw, W., J., Bizzarro, S., Muris, J., Su, N., Nicu, E. A., Nazmi, K., Bikker, F. J. & Loos, B. G. (2019) ‘A rapid, non‐invasive tool for periodontitis screening in a medical care setting’. BMC Oral Health, 19 (1), pp. 87.	No relevant data
Wadia, R. (2020) ‘The Updated Periodontal Classification: Answers to 10 Common Questions’. Primary Dental Journal, 8 (4), pp. 18–21.	No relevant data
Wadia, R., Walter, C., Chapple, I. L. C., Ower, P., Tank, M., West, N. X., Needleman, I., Hughes, F. J., Milward, M. R., Hodge, P. J. & Dietrich, T. (2019) ‘Periodontal diagnosis in the context of the 2017 classification system of periodontal diseases and conditions: Presentation of a patient with periodontitis localised to the molar teeth’. British Dental Journal, 226 (3), pp. 180–182.	No relevant data
Walter, C., Chapple, I. L. C., Ower, P., Tank, M., West, N. X., Needleman, I., Hughes, F. J., Wadia, R., Milward, M. R., Hodge, P. J. & Dietrich, T. (2019) ‘Periodontal diagnosis in the context of the BSP implementation plan for the 2017 classification system of periodontal diseases and conditions: Presentation of a pair of young siblings with periodontitis’. British Dental Journal, 226 (1), pp. 23–26.	No relevant data
Walter, C., Chapple, I. L. C., Ower, P., Tank, M., West, N. X., Needleman, I., Wadia, R., Milward, M. R., Hodge, P. J. & Dietrich, T. (2019) ‘Periodontal diagnosis in the context of the BSP implementation plan for the 2017 classification system of periodontal diseases and conditions: Presentation of a patient with severe periodontitis following successful periodontal therapy and supportive periodontal treatment’. British Dental Journal, 226 (6), pp. 411–413.	No relevant data
Walter, C., Chapple, I. L. C., Ower, P., Tank, M., West, N. X., Needleman, I., Wadia, R., Milward, M. R., Hodge, P. J. & Dietrich, T. (2019) ‘Periodontal diagnosis in the context of the BSP implementation plan for the 2017 classification system of periodontal diseases and conditions: Presentation of a patient with a history of periodontal treatment’. British Dental Journal, 226 (4), pp. 265–267.	No relevant data
Walter, C., Ower, P., Tank, M., West, N. X., Needleman, I., Hughes, F. J., Wadia, R., Milward, M. R., Hodge, P. J., Chapple, I. L. C. & Dietrich, T. (2019) ‘Periodontal diagnosis in the context of the 2017 classification system of periodontal diseases and conditions: Presentation of a middle‐aged patient with localised periodontitis’. British Dental Journal, 226 (2), pp. 98–100.	No relevant data
West, N., Chapple, I., Claydon, N., D'Aiuto, F., Donos, N., Ide, M., Needleman, I. & Kebschull, M. (2021) ‘BSP implementation of European S3 – Level evidence‐based treatment guidelines for stage I‐III periodontitis in UK clinical practice’. Journal of Dentistry, 106, pp. 103562.	No relevant data
West, N., Chapple, I., Culshaw, S., Donos, N., Needleman, I., Suvan, J., Nibali, L., Patel, A., Preshaw, P. M. & Kebschull, M. (2024) ‘BSP implementation of prevention and treatment of peri‐implant diseases – The EFP S3 level clinical practice guideline’. Journal of Dentistry, pp. 104980.	No relevant data
Yanaranci, S., Laosrisin, N., Sriprasertsuk, A., Panrin, P. & Nantakeeratipat, T. (2024) ‘The Association of Maternal Periodontal Diseases in the Postpartum Period with Preterm Low Birth Weight’. The Journal of Contemporary Dental Practice, 25 (2), pp. 99–106.	No relevant data

## Appendix 5 Part 1—Data Extraction Form

**Table:**

Review ID	Title	First author	Corresponding author	Corresponding author email	DOI	Year of publication	Country of study	Study aims and/or objectives	Ethical approval obtained?	PPI input?
Conflict of interests reported	Study design	Number of participants *(in final analysis)*	Diagnostic test outcome reported	Statistical analysis used	Study result (effect size)	p‐value	Confidence intervals (95% CI)	Conclusion	Appraisal tool required	

## Appendix 6 Part 2—Data Extraction Form

**Table:**

Review ID	Title	Country of study	Type of paper (primary research; quasi experimental; evidence synthesis; narrative or opinion piece etc.)	PPI input? (Y or N)	Conflict of interests reported	Study design (if appropriate)
Identified facilitator themes	Facilitator corresponding quotes	Identified barrier themes	Barrier corresponding quotes	Other comments		
