# Supplementary material for: Evaluating the Performance and Implementation of the 2018 Classification of Periodontal Diseases: A Systematic Review and Survey
Source: J Clin Periodontol. 2025 May 7;52(Suppl 29):34–57. doi: 10.1111/jcpe.14170 (PMC12286650; doi:10.1111/jcpe.14170)
Supplement: Supplementary file 3 — Supporting Information S3. Appendix 13: Part 2—Code book. [file JCPE-52-34-s003.docx]

**Appendix 13: Part 2 – Code book**

| Theme | Primary Codes | Sample Quotes |
| --- | --- | --- |
| **Factors that facilitate the implementation of the 2018 Classification** | Clinical factors   - Evidence based - Individualised approach to diagnosis and treatment planning - Risk factor profiles - Grade modifiers allow risk-based treatment plan - Grading promotes prevention and periodontal maintenance - High sensitivity for detecting early disease (allowing preventative management) - Promote early referral - Promotion of interdisciplinary working - `Predictive value for tooth loss - Prognostic value | This case study exemplifies the means by which the new system looks at contributing factors to estimate the aggressiveness of the disease and takes into account individual factors that will likely impact the response to treatment and its desired outcomes (Aboalsaud et al., 2023).  In addition, the new staging and grading system is based on the medical model. Thus, it is purposefully designed to drive interprofessional collaboration to mitigate systemic and behavioural factors that impact disease manifestation, progress and response to treatment (Aboalsaud et al., 2023).  The structure of the new staging and grading system promotes individualized, patient-centred care, which respondents valued as important to the provision of optimal oral health care (Aboalsaud et al., 2023).  This enables the clinician to give individualized diagnosis and tailor made treatment plans for every patient (Babay et al., 2019).  An evidence-based classification of periodontal and peri-implant diseases provides clarity and direction for the care of patients, as well as offering improved means of communication with colleagues (Claydon et al., 2022).  ...that each patient should carry a working diagnosis of their periodontal status. In consequence, the 2017 classification empowers clinician-patient partnerships to direct personalised pathways of oral care towards ‘well-defined therapeutic outcomes’ (Claydon et al., 2022).  At patient level, it allows well-defined therapeutic outcomes to be established. It also acts as an objective target for clinicians, which empowers patients to contribute to treatment planning and recall interval. At population level, it allows for surveillance of periodontal status (Claydon et al., 2022).  ....for the first time has highlighted the importance of %BOP as a true, validated and tangible marker of disease activity which is readily understood by patients (Claydon et al., 2022).  The high sensitivity value underlines the capacity of the new case definition system to detect periodontitis, including mild periodontal destructions, which greatly favors early treatment (Costea et al., 2022).  The 2018 classification of periodontal diseases seems to have a predictive accuracy for tooth loss in cases with high severity (El Sayed et al., 2022).  In the new classification, periodontitis is defined as CAL at two non-adjacent teeth. Even though the case definition does not stipulate a threshold (which might avoid misclassification), any numerical CAL value must be defined epidemiologically. Compared to the AAP/CDC definition (a CAL >= 3 mm), Stage I represents early periodontitis, which is difficult to distinguish from advanced gingivitis. Thus, the decrease in the CAL threshold of the new classification increased the prevalence of periodontitis (Germen et al., 2021).  Potential advantages of the new classification include a reported ability to reliably predict the incident tooth loss and a lower susceptibility to bias as compared to the Centers for Disease Control and Prevention/ American Academy of Periodontology (CDC/AAP) classification when partial-mouth recording protocols are used (Holtfreter et al., 2024).  Moreover, the assessment of grade involves the identification of common risk factors for periodontitis and facilitates interdisciplinary cooperation (Holtfreter et al., 2024).  *"Grade modification considering the effects of these parameters in smokers and diabetic individuals provides clinicians the data needed to develop a higher risk-based treatment plan." "[...] while no correlation was observed between the radiographic grade and the modified grade in smokers and diabetic patients, increasing the grade score for these individuals in accordance with the 2017 classifications provides clinicians the opportunity to develop a risk-based treatment plan in order to better prevent recurrent periodontal destruction" (Karaaslan et al., 2021).*  Staging relies on the severity and extent of periodontitis at presentation but also introduces the practical and individualized dimension of case complexity (Miyamoto et al., 2019).  Proper grading estimates environmental and genetic risk interactions for future risk, as in this case, in which the patient has the potential to progress at a similar rate as the mother (Miyamoto et al., 2019).  Taking family history of periodontal disease in close relatives when reviewing dental history with the patient is significant in the new classification system (Miyamoto et al., 2019).  Understanding the grading of periodontal disease has a significant impact on the prognosis of dentition (Miyamoto et al., 2019).  Moreover, grading is critical for planning prevention of periodontal disease and indicates the need for meticulous periodontal maintenance (Miyamoto et al., 2019).  Teaching of the new classification and wide adoption of staging and grading could increase early referral, early periodontal treatment intervention leading to decreased tooth loss, and have a potential effect on overall health improvement and wellness (Miyamoto et al., 2019).  *Given the expanding body of literature, this new classification seems to have received a largely positive response from the dental community, and it has been introduced as the basis for clinical treatment practice guidelines by the EFP and epidemiological investigations around the world. (Raittio et al., 2024)*  The 2018 classification is assumed to have prognostic value towards future treatment successes and the required treatment efforts, allowing individualized treatment planning. If so, it should also reflect treatment costs during active periodontal treatment (APT), but also long term during supportive periodontal treatment (SPT). This would allow to guide patients and other decision-makers towards the potential cost effectiveness of periodontal treatment and tooth retention according to different diagnoses (Schwendicke et al., 2020).  Based on data from a cohort of compliant periodontitis patients we demonstrate that costs were associated with grade, but not stage; a similar association was found to tooth loss in this cohort before (Graetz et al., 2019) (Schwendicke et al., 2020).  Second, we found tooth loss to be associated with stage and grade (more teeth were lost in stage IV than III and grade C than B), while costs were rather driven by grade (higher costs in grade C than B) (Schwendicke et al., 2020). |
|  | Factors for epidemiological studies   - Use with full mouth partial diagnostic protocols - Sensitivity for detecting disease in partial diagnostic protocols - Correlation with tooth loss - Correlation with cost effectiveness - Inclusion of mid buccal and mid lingual sites conveys entire periodontal condition | Our results confirmed that the new 2018 classification outperforms the 2012 classification regarding the diagnosis of periodontitis and its staging on full-mouth PRPs (Botelho et al., 2020).  The inclusion of central surfaces (mid-buccal and mid-lingual sites) in the 2018 case definition has endowed it with a holistic view of the periodontal situation. In other words, by considering all circumferential sites we increase the likelihood of correctly diagnosing periodontitis, rather than the 2012 classification that only uses interproximal locations (maximum of four sites) (Botelho et al., 2020).  On the one hand, the new 2018 EFP/AAP case definition is a reliable tool in depicting patients’ characteristics, disease progression and tooth loss. On the other hand, our findings emphasize its reliability on future epidemiological studies using PRPs, considering that more surveys are warranted to improve surveillance of periodontitis, a pandemic disease with worldwide prevalence and worrisome socio-economic impact (Botelho et al., 2020).  In indexes with a lower number of teeth, the current 2018 case definition endowed CPITN has a more reliable tool in both detecting and staging periodontitis, comparing to the 2012 scenario. For the “Ramfjord teeth”, the 2018 classification provided slight surveillance improvements, though it was the index with less favorable performance (Botelho et al., 2020).  Potential advantages of the new classification include a reported ability to reliably predict the incident tooth loss and a lower susceptibility to bias as compared to the Centers for Disease Control and Prevention/ American Academy of Periodontology (CDC/AAP) classification when partial-mouth recording protocols are used (Holtfreter et al., 2024).  Based on data from a cohort of compliant periodontitis patients we demonstrate that costs were associated with grade, but not stage; a similar association was found to tooth loss in this cohort before (Graetz et al., 2019) (Schwendicke et al., 2020).  Second, we found tooth loss to be associated with stage and grade (more teeth were lost in stage IV than III and grade C than B), while costs were rather driven by grade (higher costs in grade C than B) (Schwendicke et al., 2020).  In 2012, the Centers for Disease Control (CDC) with the AAP proposed a standard case definition for surveillance periodontitis based on measurements of periodontal probing depth (PPD) and clinical attachment loss (CAL) at interproximal sites. Ever since this case definition has been widely accepted and applied both in epidemiological and clinical research. In the new 2018 classification, in addition to the interproximal sites, mid-buccal and mid-lingual sites were also considered. Comprehensively, this addition reinforces this new classification with a potential improved ability to transmit the entire periodontal condition. (Botelho et al., 2020) |
|  | Implementation strategies   - Use of flowcharts to aid classification and improve accuracy | To overcome these problems, simple and rapid decision flowcharts have been developed and proposed not only to facilitate the performance of fast and accurate periodontitis staging and grading, but also to minimize confusion and inconsistent diagnoses (Ertas et al., 2023).  *Flowcharts offer a straightforward approach to improving the accuracy of diagnosing periodontal conditions in a time-effective manner (Parsegian et al., 2021).*  *The major benefit of the proposed flowcharts is to aid clinicians to a simple and quick screening so a correct periodontal diagnosis can be obtained. In contrast to the other decision tree, the proposed flowchart provides criteria to differentiate periodontal health, gingivitis, and periodontitis according to the 2018 case definition in the same flowchart, which makes it easier to follow. Additionally, not all clinical parameters are needed to make a periodontal diagnosis in every case. In this flowchart, clinical AL measurement may be skipped in some cases or it can be done only when necessary. However, this flowchart only focuses on plaque-induced periodontal diseases. Attachment loss or bone loss from non-periodontitis causes will be considered as “no RBL/clinical AL” to avoid false positive in a diagnosis of periodontitis (Sutthiboonyapan et al., 2020).* |
|  | Local implementation strategies   - BSP-I easy to implement in practice - BSP-I flowchart - BSP-I webinars - BSP-I improved communication with colleagues - BSP-I simplification of the classification process - BSP-I no negative impacts on class allocation nor prognostic performance - UK, USA, Holland and Ireland have produced local implementation strategies - Slight variations in their interpretation of the classification - University teaching strategies | The authors consider this to indicate that application of the BSP implementation of the 2017 classification is attainable in the general dental practice operational framework, within a one-year timeframe (Claydon et al., 2022).  Importantly, this study has shown that implementation of the classification is possible in clinical practice, with 92% of patients being diagnosed based on the new classification. This may be in part due to the great efforts made by the BSP to widely distribute educational material regarding the classification, aided by the uptake of social media in the dental profession. Such an implementation was arguably impossible at the time the 1999 classification was released (Claydon et al., 2022).    *The main rationale behind abridging the 2017 WWC by the BSP-I was to simplify the classification process for clinicians in practice, especially general practitioners and dental students. This could make treatment outcomes more predictable and improve our ability to share findings with our patients. A few examples of cases that demonstrate the practical implementation of the BPS-I were published recently. More importantly, the results presented in this manuscript demonstrated that the BSP’s pragmatic approach through implementing a reductionist model of the original 2017 WWC neither affected the class allocation nor the prognostic performance of the system. This should be considered as the most significant finding in terms of practicality of implementing this classification in general dental practice (Dukka et al., 2022).*  The British Society of Periodontology have produced a flow chart for implementing the 2017 classification in clinical practice11 and Figure 1 outlines an example of how the 2017 classification can be applied. Numerous recent publications provide other examples detailing the application of this new classification (Graham and Turner, 2020).  One example of such dissemination effort was the British Society of Periodontology webinars. These webinars aimed at explaining the contents of the four working groups that drew up the design of the new classification system for UK dentists and dental hygienists on the new classification. This type of effort is commendable because the integration of information for determining the stage and grade of periodontitis may appear challenging in the beginning (Gandhi et al., 2022).    The department holds monthly consensus training meetings for periodontics residents and faculty members to help maintain consistency while teaching pre-doctoral students (Gandhi et al., 2022).  Each country has its own requirements for the 2017 classification due to variations in healthcare needs within different healthcare systems. Therefore, there is a need to implement the world classification of periodontology to suit their own rules and regulations. Several countries including the United Kingdom, United States, Holland and Ireland have recognised and actioned on this point, each with slight variations in their interpretation of the classification (Jayawardena et al., 2021).  There has been ongoing work by the BSP including a series of webinars and case series to provide education regarding the implementation of the 2017 classification (Jayawardena et al., 2021).  Further guidance on the UK implementation of the 2017 classification can be found through the free to access BSP resources presented online (Jayawardena et al., 2021). |
|  | Technological implementation strategies   - App-based classifiers - Use of machine learning to aid classification - Computer aided diagnostics - Deep learning (AI) to assess radiographic bone loss - Digital tools to aid classification - Automated computer algorithms to monitor treatment outcomes and develop prediction models - Electronic health records based algorithm | The machine learning-based decision system presented herein can facilitate periodontal diagnoses despite its current limitations (Ertas et al., 2023).  Artificial intelligence intends to reproduce the cognitive processes of the human being and obtain the same result, in this case, the determination of the diagnosis of a disease with that produced by the clinician, with more accuracy and shorter time (Fidyawati et al., 2024).  Periodontal disease staging is a new and challenging situation for most physicians because it contains many parameters. Computer-aided diagnostic (CAD) provides a second opinion to dentists in diagnosing and classifying pathologies and diseases (Guler Ayyildiz et al., 2024).  The study stated that the convolutional neural network (CNN) method is similar to dentists' ability to assess radiographically periodontal bone loss and, therefore, is a promising assistant diagnostic tool. In a different study, the deep CNN based autonomous transfer learning (TL) method and pre-knowledge of lesion correlation were used to detect bone loss in panoramic radiographs. The model proposed in the analysis results has been reported to perform better than expert dentists and can correctly detect radiographic bone loss (Guler Ayyildiz et al., 2024).  The study, which contains an automatic and hybrid method for staging CNN and CAD synthesis and periodontitis, reported that deep learning (DL) shows high accuracy and perfect reliability in diagnosing periodontal bone loss and in staging periodontitis (Guler Ayyildiz et al., 2024).  *The DL model provides an accurate and reliable alveolar bone level measurement, RBL stage assignment, and preliminary periodontal diagnosis based on periapical radiographs. DL can be utilized as a tool to assist clinicians in diagnosing periodontitis in the clinic and further making the proper treatment plan (Lee et al., 2022).*  *Most (80%) DHs were positive about a digital tool and believed that it could both supplement and support work with the classification system. Digital technology offers tools that could facilitate and improve decision-making and by extension, optimize the patient's care. Under the new classification system, the individual clinician's input and reasoning are still important and should be emphasized when developing a digital tool for assisting in classification (Malmqvist et al., 2024).*  *Our pilot study shows that the PerioClassApp may reliably transform the manual classification process to a more standardized, user friendly digital evaluation of periodontal cases (Meir et al., 2022).*  *The automated approaches generated in this study can be utilized to automatically document patients’ PD diagnoses because of the fragmented reporting of diagnosis. Next, this phenotype approach could be utilized to first improve the completeness of the EDR data which then can be utilized to study PD. For example, this approach can be utilized to examine the long-term periodontal treatment outcomes and to develop prediction models (Patel et al., 2022).*  *Participants said that when they used the app, the questions were very specific about periodontal parameters that define the periodontal condition and therefore improved their understanding of the new classification system. In contrast, during mental diagnosis, they depended on previous knowledge and had to look up the definition of each periodontal condition. While this would be normal in the learning curve of students, we consider that PerioSmart could be a helpful tool for learning and acquiring the basis of periodontal diagnosis for future practice and help dental students and professionals familiarize themselves with the general rules of the new classification of periodontal diseases (Sanchez-Otalvaro et al., 2022).* |
| **Factors acting as barriers to the implementation of the 2018 Classification** | Practical barriers to implementation   - Lack of time - Learning a new classification - Complexity of the classification system - Subjectivity - Inter rater reliability - Classifying borderline cases | The main barriers reported were lack of faculty buy-in, lack of information and CE courses covering the new staging and grading system and no time in the curriculum for implementation. These barriers could be attributed to a lack of faculty calibration and insufficient faculty development workshops (Aboalsaud et al., 2023).  Another significant barrier was the challenge of introducing the new system while also still teaching the 1999 classification system because the NBDHE will not be testing on the new staging and grading system until the fall of 2021. Additional barriers reported included (a) the COVID-19 pandemic, (b) lack of time and (c) faculty calibration. (Aboalsaud et al., 2023)  However, clinicians have had difficulties adopting and applying this classification in daily practice, and many clinicians have complained about difficulties in determining the stage and grade of periodontitis because of the presence of many clinical and radiographic factors that need to be considered in the current classification and in periodontal screening studies. (Ertas et al., 2023)  Most dentists will be familiar with the publication of the updated disease classification; however, many have found the adaptation to this new classification challenging. (Graham and Turner, 2020)  Periodontal disease staging is a new and challenging situation for most physicians because it contains many parameters. Computer-aided diagnostic (CAD) provides a second opinion to dentists in diagnosing and classifying pathologies and diseases. (Guler Ayyildiz et al., 2024)  The new periodontal disease classification is a complex process that includes staging, grading, and subheadings. Especially during staging, there is a 1-2mm difference to differentiate radiographic bone loss in stage 1 and stage 2. Again, to evaluate the difference between stage 3 and stage 4, different criteria such as periodontal tooth deficiency, furcation, occlusion, drifting, and flaring should be included in DL methods. (Guler Ayyildiz et al., 2024)  Additionally, as there are several subjective factors that go into formulating a periodontal diagnosis and treatment plan; there is a high chance of variability in clinical decision making, especially with the many changes from the 1999 classification. (Gandhi et al., 2022)  Although consensus training programs for dental students have been introduced, consistency in clinical decision-making among dental students is less than ideal. (Gandhi et al., 2022)  In our study, the percentage of correct responses for periodontal diagnosis was low compared to treatment planning. The lower rate of correct responses in periodontal diagnosis could be attributed to the new classification being more extensive and detailed, compared to the previous 1999 classification. (Gandhi et al., 2022)  It may therefore become more challenging for dental students to consider all the factors involved in an accurate periodontal diagnosis. (Gandhi et al., 2022)  Although accurate staging and grading of the disease are important for the comprehensive management of periodontal disease, the presence of overlapping criteria may increase the difficulty for dental students to differentiate between stages III and IV. (Gandhi et al., 2022)  *Borderline cases require clinicians to additionally rely on their clinical judgement to overcome strict algorithmic assessments outside of the parameters of the general guidelines in order to arrive at an appropriate diagnosis and treatment plan. (Steigmann et al., 2021)* |
|  | Problems implementing in epidemiological studies   - Lack of appropriate radiographs - Need for full mouth inspection - Increased reliance on need for full mouth radiographic examination - Less favourable performance with Ramfjord teeth - Changing case definition limiting comparisons between studies e.g. evidence synthesis - Worries re: over estimating disease prevalence - Misdiagnosis due to misinterpretation - New case definition has no lower limit for CAL – increased prevalence of periodontitis - Difficulties determining reason for previous tooth loss - Difficulties determining gingivitis from early periodontitis - Complexity of classification - Radiographic grade may be wrong if worst tooth already extracted - Complexity of data collection | However, the grading is less reliable without a full‐mouth radiographic assessment, which is not routinely conducted or recommended for epidemiological studies. Previous studies either adopted staging only for population‐based data (Botelho et al., 2020) or acknowledged the challenges of using the full matrix of staging, grading, and descriptors of distribution (Germen et al., 2021; Ndjidda Bakari et al., 2021). (Alawaji et al., 2022)  Furthermore, the 2012 and 2018 case definitions demand circumferential full-mouth inspection, which in large surveys and epidemiological studies it is often difficult to conduct, and time and labour intensive. (Botelho et al., 2020)  For the “Ramfjord teeth”, the 2018 classification provided slight surveillance improvements, though it was the index with less favorable performance. (Botelho et al., 2020)  Both the 2012 CDC/AAP and the 2018 EFP/AAP case definition systems use a combination of parameters—clinical attachment loss (CAL) and probing depth (PD)—to define periodontitis, but the threshold values and their combinations are different. This can result in significant differences in periodontitis identification and prevalence values, creating confusion surrounding the associative relationships with risk factors or general diseases, thus hindering the direct comparison between studies. (Costea et al., 2022)  This observation has been found particularly relevant in the context of assessing the impact of periodontitis in patients with various systemic diseases, given that obtaining population groups numerically adapted to the research purposes is extremely challenging, and the results of multiple studies with a uniform design, including a similar case-definition, need to be corroborated in order to increase the reliability of the results. (Costea et al., 2022)  On the other hand, it is possible that incipient cases may be also overestimated by the current classification system due to errors intrinsic to the measurement of clinical periodontal parameters of 1 mm [39,59] induced by many technical variables. Unlike the 2012 CDC/AAP case definition system, the new one not only lowers the CAL diagnostic threshold, but also considers mid-buccal and mid-oral periodontal measurements, which may increase the prevalence of periodontitis. However, this may lead to a miscalculation of the periodontitis case if the aetiology of buccal and oral CAL loss is incorrectly provided by the evaluator. (Costea et al., 2022)  *The 2018 EFP/AAP classification allows comprehensive assessment; however, it requires full-mouth inspection, which can be time- and labor-intensive in population based surveys and epidemiological investigations. In the present study, the general population was the target, approximately 70% of the participants had their severity upgraded by the 2018 EFP/AAP classification compared to the corresponding cluster. However, concerning “periodontitis cases,” the 2018 EFP/AAP classification showed a higher level of consistency with the clustering analysis. These findings suggest that the 2018 EFP/AAP classification better distinguishes “periodontitis cases” than the general population. We thus presume that the 2018 EFP/AAP classification is sensitive to identifying “periodontitis cases” and tends to be more suitable for clinical usage than epidemiological surveillance purposes. In addition, the 2012 CDC/AAP definition is more effective in identifying a population free from periodontitis. It is therefore expected to be continually used for surveillance in the future. (Du et al., 2023)*  In the new classification, periodontitis is defined as CAL at two non-adjacent teeth. Even though the case definition does not stipulate a threshold (which might avoid misclassification), any numerical CAL value must be defined epidemiologically. Compared to the AAP/CDC definition (a CAL >= 3 mm), Stage I represents early periodontitis, which is difficult to distinguish from advanced gingivitis. Thus, the decrease in the CAL threshold of the new classification increased the prevalence of periodontitis. (Germen et al., 2021)  Reasons for previous tooth loss is one of the factors to consider in the new classification and understandably presents dentists with challenges as patients may have had multiple previous dentists and not be able to accurately recall the reasons for the loss of their previous teeth. (Graham and Turner, 2020)  In contrast, the application of the 2018 classification to epidemiological surveys presents some significant challenges. First, it is usually not possible to accurately ascertain the reasons for tooth loss. Thus, the number of missing teeth due to periodontitis cannot be reliably determined. Notably, the few epidemiological studies applying the 2018 system so far have largely excluded tooth loss data from the assessment of stage. However, most of those few studies that included tooth loss attributable to periodontitis in the assessment of stage have predominantly used assumptions rather than primary data. Second, complexity factors (including the presence of horizontal or vertical bone loss [BL], moderate or severe ridge defects, masticatory dysfunction and secondary occlusal trauma) are not routinely recorded in epidemiological surveys, resulting in a potential underestimation of the true prevalence of stage III or IV periodontitis. Consequently, a correct assignment of—and differential classification between—stage III and IV periodontitis based on clinical periodontal data alone may have been significantly impaired in the above studies, given the lack of information on periodontitis-related tooth loss and other complexity factors. Third, it appears that previously published epidemiological surveys using the 2018 classification encountered additional difficulty in the distinction between gingivitis and periodontitis, as none of them reported the presence of gingivitis cases despite the inclusion of young individuals. Lastly, a concrete framework on how to apply the 2018 scheme to epidemiological data has not been provided in the original publication or the consensus reports allowing discrepancies due to different interpretations. (Holtfreter et al., 2024)  "The radiographic grade measurement is determined according to the bone loss of the worst tooth in the mouth, but the worst tooth may have already been extracted. Thus, the radiographic grade may be understated. Modifying the grade independently of the radiographic grade avoids this and allows the patients to be assigned to a higher risk class to encourage more effective control." (Karaaslan et al., 2021)  *Second, staging and grading using the new periodontal classification require detailed records of a patient's periodontal state. Some factors were not included in the interview and dental examination during the survey. In terms of staging, these factors include radiographic bone loss, reasons for tooth loss and the need for complex rehabilitation. For grading, these factors include changes in radiographic bone loss over time, the amount of destruction (due to biofilm deposits), diabetes and specific biomarkers. (Ke et al., 2023)*  *Several DHs stated that the new system was too time-consuming, that it caused stress, that their knowledge was inadequate and that they, therefore, considered it too difficult to use. (Malmqvist et al., 2024)*  Some of us have recently demonstrated that the new classification system did not differ from previous systems in its presuming approach to case definitions and no evidence has been presented for the superiority of the new classification system over previous models for the prediction of clinically important outcomes. (Raittio et al., 2024)  Furthermore, its development was not based on a balanced assessment of the potential benefits and harms associated with its implementation. It therefore remains unknown whether the use of that system provides more net benefits to patients and to the community than previous classification systems. What is evident, however, is that the use of this classification for epidemiological purposes results in prevalence estimates that do not align well with the public health importance of periodontitis, and overdiagnosis is likely to be a considerable problem leading to an unfavourable benefits-to-  harms ratio for major sections of any given population. (Raittio et al., 2024)  In line with all other epidemiological studies using the criteria  of the new AAP/EFP periodontitis classification system, the studies  emerging from Norway have indicated exorbitantly large periodontitis estimates amongst Norwegians. (Raittio et al., 2024)  This may be a one explanation for the considerable increase in the public subsidy for the cause-related treatment of periodontitis from 21.1 million Euros in 2013 (before the introduction of the new classification system) to 53.6 million Euros in 2022 (after the introduction), representing an increase of 83%. These figures are deflated by the consumer price index for dental services. The increase without adjusting for inflation is 154%. This raises the rather pertinent question of whether this increment reflects a recent outbreak of periodontitis amongst Norwegians, or whether the apparent epidemic has been created by the dental profession itself through the implementation of the new classification system. (Raittio et al., 2024)  Indications that the latter is the case is found in the observation that implausibly high disease prevalence and workforce requirement estimates accompanied by low actual spending on periodontal care are not new phenomena and has occurred with earlier approaches for estimating periodontal treatment needs, the Community Periodontal Index of Treatment Needs and Russell's Periodontal Index. (Raittio et al., 2024)  In our view, these Norwegian data support the criticisms raised against the 2017 AAP/EFP periodontitis classification system in that it is likely to lead to overdiagnosis and overtreatment and will shift the benefits-to-  harms ratio for periodontitis treatment in a negative direction for both patients and for society at large. (Raittio et al., 2024)  ... the only practical way forward involves a re-evaluation of disease definitions (i.e. a revision of the new periodontitis classification system) and the treatment thresholds, duration and frequency (i.e. the clinical treatment practice guidelines  for periodontitis), aiming to attain levels that are both practically and financially feasible and relevant for patient-important outcomes. In the UK, an alternative approach is taken and the EFP guidelines are now implemented so that more resources are used on ‘engaging patients’  who represent adequate oral hygiene and risk factor control in re-evaluation, whereas the ‘non-engaging patients’ are recommended to receive palliative periodontal care. In our view, people and societies who apply and promote such approaches have the burden of proof whether this approach results in better patient-important outcomes than alternative, less invasive and resource-demanding approaches for the whole population, irrespective of engagement. (Raittio et al., 2024)  Further data which may be required for fully applying the new classification, like the presence of plaque, masticatory dysfunction, bite collapse, drifting or flaring were not consistently available and hence not used for the present study. (Schwendicke et al., 2020) |
