# Supplementary material for: Evaluating the Performance and Implementation of the 2018 Classification of Periodontal Diseases: A Systematic Review and Survey
Source: J Clin Periodontol. 2025 May 7;52(Suppl 29):34–57. doi: 10.1111/jcpe.14170 (PMC12286650; doi:10.1111/jcpe.14170)
Supplement: Supplementary file 4 — Supporting Information S4. Appendix 14: Part 2—Survey. [file JCPE-52-34-s001.docx]

**Appendix 14: Part 2 – Survey**


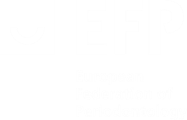


EFP Feedback Survey

*The EFP is undertaking a series of Systematic Reviews as part of the XX European Workshop on Periodontology entitled : “Periodontal diagnosis – From advances in technologies to the 2018 classification”. As part of this process, we are seeking your feedback on the 2018 Classification with a particular focus on its use in Periodontal Diseases. We are grateful for your time spent completing this short survey, that should take no longer than 5-minutes to complete. Please note, all responses are anonymous unless you give us your contact details (and therefore consent to be contacted) seeking further information on the 2018 Classification. If you do provide your contact details, they will be removed from our encrypted database once you have been sent the relevant information. The closing date for the survey is the 19th July. The EFP we would really value your input.*

1. **Country**
2. **How old are you?**

Under 18

18-24

25-34

35-44

45-54

55-64

Over 65 years

1. **What profession best describes yourself?**

Undergraduate student Dentist

Undergraduate student Hygienist or Dental Therapist

Hygienist or Dental Therapist

General dental practitioner or dentist with a special interest

Specialist in Periodontology

Other dental specialist

Full time academic

Part time academic (plus private practice)

Full time Hospital or government service

Part time hospital or government service (plus private practice)

Prefer not to say

Other

1. **Are you aware of the 2018 Periodontal Classification**

Yes

No

1. **Please enter your email if you would like to be sent more information, otherwise leave blank**
2. **Is the 2018 periodontal classification used by your insurance carriers and/or your state healthcare system ?**

Yes

No

Unsure

1. **Has your national society produced advice for implementing the 2018 periodontal classification?**

Yes

No

Don't know

1. **Do you use the 2018 periodontal classification?**

Yes

Sometimes

No

1. **What influences you to use the 2018 periodontal classification?**
2. **If no, which periodontal classification are you using?**
3. **What could be changed to support you in using the 2018 periodontal classification?**
4. **How easy is it to use the 2018 periodontal classification? (**1 = not easy at all, 5 = very easy)

1

2

3

4

5

1. **Does the 2018 periodontal classification meet your requirements?**

Yes

Somewhat

No

1. **Have you encountered difficulties when staging and grading?**

Yes

No

1. **If so, what aspect is difficult?**
2. **What would you like to see in the 2018 periodontal classification?**
3. **Do you think the 2018 periodontal classification is an improvement on the previous classification?**

Yes

No

1. **If yes, why is it an improvement?**
2. **If no, why?**
